# Conjugation of Diclofenac with Novel Oleanolic Acid Derivatives Modulate Nrf2 and NF-κB Activity in Hepatic Cancer Cells and Normal Hepatocytes Leading to Enhancement of Its Therapeutic and Chemopreventive Potential

**DOI:** 10.3390/ph14070688

**Published:** 2021-07-17

**Authors:** Maria Narożna, Violetta Krajka-Kuźniak, Barbara Bednarczyk-Cwynar, Małgorzata Kucińska, Robert Kleszcz, Jacek Kujawski, Hanna Piotrowska-Kempisty, Adam Plewiński, Marek Murias, Wanda Baer-Dubowska

**Affiliations:** 1 Department of Pharmaceutical Biochemistry, Poznan University of Medical Sciences, 60-781 Poznan, Poland; maria.narozna@ump.edu.pl (M.N.); vkrajka@ump.edu.pl (V.K.-K.); kleszcz@ump.edu.pl (R.K.); 2 Department of Organic Chemistry, Poznan University of Medical Sciences, 60-780 Poznan, Poland; bcwynar@ump.edu.pl (B.B.-C.); jacekkuj@ump.edu.pl (J.K.); 3 Department of Toxicology, Poznan University of Medical Sciences, 60-631 Poznan, Poland; kucinska@ump.edu.pl (M.K.); hpiotrow@ump.edu.pl (H.P.-K.); marek.murias@ump.edu.pl (M.M.); 4 Centre for Advanced Technologies, Adam Mickiewicz University, 61-614 Poznan, Poland; adam.plewinski@amu.edu.pl

**Keywords:** diclofenac, oleanolic acid derivative conjugates, NF-κB, Nrf2, HepG2 cells, THLE-2 cells, HCC, inflammation, apoptosis, reactive oxygen species, NOD/SCID mice

## Abstract

Combining NSAIDs with conventional therapeutics was recently explored as a new strategy in cancer therapy. Our earlier studies showed that novel oleanolic acid oximes (OAO) conjugated with aspirin or indomethacin may enhance their anti-cancer potential through modulation of the Nrf2 and NF-κB signaling pathways. This study focused on the synthesis and biological evaluation of four diclofenac (DCL)–OAO derivative conjugates in the context of these pathways’ modification and hepatic cells survival. Treatment with the conjugates **4d**, 3-diclofenacoxyiminoolean-12-en-28-oic acid morpholide, and **4c**, 3-diclofenacoxyiminoolean-12-en-28-oic acid benzyl ester significantly reduced cell viability in comparison to the DCL alone. In THLE-2, immortalized normal hepatocytes treated with these conjugates resulted in the activation of Nrf2 and increased expression in SOD-1 and NQO1, while the opposite effect was observed in the HepG2 hepatoma cells. In both cell lines, reduced activation of the NF-κB and COX-2 expression was observed. In HepG2 cells, conjugates increased ROS production resulting from a reduced antioxidant defense, induced apoptosis, and inhibited cell proliferation. In addition, the OAO morpholide derivative and its DCL hybrid reduced the tumor volume in mice bearing xenografts. In conclusion, our study demonstrated that conjugating diclofenac with the OAO morpholide and a benzyl ester might enhance its anti-cancer activity in HCC.

## 1. Introduction

Hepatocellular carcinoma (HCC) remains the most frequent primary liver cancer and is a leading cause of cancer-related deaths. Chronic inflammation resulting mainly from viral hepatitis or metabolic disorders is considered the major risk factor of HCC [1]. Limited therapeutic options, particularly in the case of advanced forms of HCC, make necessary the development of new therapeutic and/or preventive strategies [2]. The key roles in the induction of inflammation involve two signaling pathways: Nrf2-ARE and NF-κB. While activation of the latter leads to transcription of genes encoding pro-inflammatory proteins, including COX-2 and iNOS enzymes, Nrf2 is responsible for cell cytoprotection and is the major cellular defense against reactive oxygen (ROS) and electrophilic species [3,4]. Overexpression of COX-2 associated with increased cell growth and invasiveness is seen in human HCC [5]. Therefore, targeting NF-κB activation is one of the possible therapeutic approaches. Usually, reduced activation and expression of NF-κB results in the opposite effect in the Nrf2 signaling pathway [6]. However, it has also been demonstrated that Nrf2, due to genetic and epigenetic alterations, is overexpressed in cancer cells, including HCC and may lead to an enhanced invasiveness potential and chemo-and radio-resistance [7].

Activation of both NF-κB and Nrf2 requires their translocation into the nucleus. Nrf2 activators enhance its binding to the Kelch-like ECH-associated protein-1 (Keap1), inducing a conformational modification that renders Keap1 incapable of promoting Nrf2 degradation and thus allowing its translocation [8]. NF-κB is sequestered in the cytoplasm through an association with its inhibitor, the IκB protein. Its activation occurs after phosphorylation by IκB kinase beta (IKK), resulting in degradation of the inhibitory subunit, enabling released NF-κB heterodimers to migrate into the cell nucleus to bind to a specific sequence encoding target genes [3].

Considering the inflammatory backgrounds of cancers such as HCC, nonsteroidal anti-inflammatory drugs (NSAIDs) are said to play a role in cancer treatment and chemoprevention. Most of these drugs act as COX-2 inhibitors, which besides their beneficial effects, i.e., decreasing the risk of certain types of cancer, are well-known for many unfavorable side effects [9].

Our earlier studies showed that oleanolic acid oxime (OAO) derivatives are potent modulators of Nrf2 and NF-κB in HCC derived HepG2 cells [10]. Moreover, conjugation with indomethacin (IND) improved that modulating impact, leading to a downregulation of these pathways. Significantly, treatment with those conjugates decreased activation of Nrf2 and NF-κB and expression of their active forms in HepG2 cells. In contrast, in normal hepatocytes, activation of Nrf2 increased, and that of NF-κB was diminished. Hence, conjugation of IND with OAO derivatives may preserve cancer cells against chemoresistance by inhibiting the Nrf2-ARE and NF-κB pathways, whilst at the same time exerting a chemopreventive impact in normal hepatocytes [11].

Diclofenac (DCL) is another commonly used NSAID, which displays various effects on the immune system, the angiogenic cascade, chemo- and radio-sensitivity, and tumor metabolism. While it is a non-selective inhibitor of both isoforms of the cyclooxygenase enzyme (COX-1 and COX-2), DCL has a preferred binding to COX-2 [12], which may explain its intermediate risk profile for gastrointestinal events in comparison to other NSAIDs [13]. Its broad-spectrum of action makes DCL one of the most potent NSAIDs in the context of cancer treatment. So far, its effect on HCC has not been well documented. Still, the co-administration of DCL and sorafenib significantly increased Bax protein expression levels leading to an increased frequency of apoptosis in HepG2 cells [14]. Moreover, a more recent study showed that DCL potentiated sorafenib-based treatment in hepatocellular cancer cells by enhancing oxidative stress [15].

**Figure 1:**
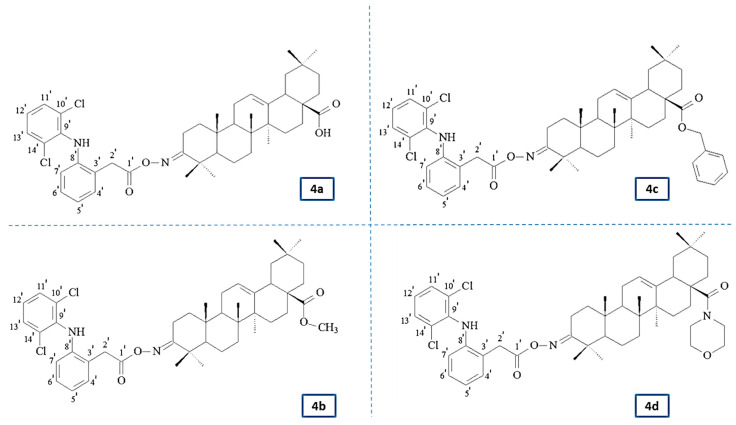
The chemical structures of the investigated novel oleanolic acid oxime derivatives with diclofenac (DCL–OAO) (**4a**–**4d**); (**4a**) 3-diclofenacoxyiminoolean-12-en-28-oic acid; (**4b**) 3-diclofenacoxyiminoolean-12-en-28-oic acid methyl ester; (**4c**) 3-diclofenacoxyiminoolean-12-en-28-oic acid benzyl ester; (**4d**) 3-diclofenacoxyiminoolean-12-en-28-oic acid morpholide.

Therefore, this study aimed to design and evaluate the effect of the newly synthesized conjugates of previously investigated OAO derivatives with DCL (Figure 1) on Nrf2 and NF-κB signaling in connection with cell cycle distribution, apoptosis, and proliferation in normal hepatocytes, and HCC cell line and tumor growth in vivo.

## 2. Results

### 2.1. Chemistry

Novel oleanolic acid oxime conjugates with DCL (DCL–OAO) (**4a**–**4d**) were obtained using previously published methods [10,16,17,18]. Scheme S1 in the Supplementary Materials presents an overview of the synthesis.

### 2.2. Spectral Characteristics of the Oleanolic Acid Oximes and Their Conjugates with Diclofenac

The chemical structures of the synthesized compounds were elucidated based on IR, ^1^H NMR, ^13^C NMR, MS/EI, and elemental analysis. Table S1 (Supplementary Materials) presents detailed spectral information about the novel DCL–OAO conjugates (**4a**–**4d**). ^1^H NMR and ^13^C NMR spectra of the novel compounds are attached in the Supplementary Materials (Figures S1–S8).

In the IR spectra of conjugates **4a**–**4d**, the presence of the NH group was confirmed by the presence of an absorption band located at about 3380 cm^−1^. The presence of aromatic systems within the DCL moiety was confirmed by the presence of an absorption band located at 3325–3335 cm^−1^. The signal observed at ν 1735–1740 cm^−1^ was assigned to the C=O group within the –COO function of the diclofenac moiety. The next characteristic absorption band, observed at ν 1720–1725 cm^−1^ was assigned to –N=C-3 of the acyloxyimino function.

The NMR analysis of conjugates **4a**–**4d** confirmed the structures of these compounds. In the ^13^C NMR spectra from the above conjugates, the signals of a double chlorinated aromatic ring within the DCL moiety (C_6_H_3_Cl_2_-NH-C_6_H_4_-CH_2_-COON=C-3) were present at about δ 142 ppm (Cq, signal intensity: 2C), 138 ppm (Cq, intensity 1C), 131 ppm (CH, intensity 2C), and 124 ppm (CH, intensity 1C). The signals derived from the second aromatic ring within the DCL moiety (C_6_H_3_C_l2_-NH-C_6_H_4_-CH_2_-COON=C-3) were observed at about 129 ppm (2xCH, both of 1C intensity), 128 ppm (Cq and CH, both of 1C intensity), 124 ppm (Cq, intensity: 1C), and 118 ppm (CH, also of intensity 1C). The acyl carbon (C_6_H_3_Cl_2_-NH-C_6_H_4_-CH_2_-COON=C-3) gave a signal present at about δ 176 ppm. The last carbons of the diclofenac system (C_6_H_3_C_l2_-NH-C_6_H_4_-CH_2_-COON=C-3) formed signals present at about δ 60 ppm.

In the ^1^H NMR spectra from the conjugates **4a**–**4d**, the protons attached to the double chlorinated aromatic (C_6_H_3_C_l2_-NH-C_6_H_4_-CH_2_-COON=C-3) formed two singlets, both of intensity 1H, observed at δ 7.36–7.34 ppm. For the derivative **4c** (benzyl ester), these signals were partially covered by signals from the aromatic ring of the benzyl moiety, so, in total, the signals from both aromatic systems (C_6_H_3_C_l2_-NH-C_6_H_4_-CH_2_-COON=C-3 and -COOCH_2_C_6_H_5_) were observed in the form of a multiplet located at δ 7.41–7.31 ppm. The next signal derived from the lat proton attached to the double chlorinated aromatic ring was observed as a triplet of a doublet, at δ 7.13–7.14 ppm. Four protons of the second aromatic ring within the diclofenac moiety (C_6_H_3_C_l2_-NH-C_6_H_4_-CH_2_-COON=C-3) formed a doublet of doublets (δ of about 7.3 ppm), two triplets (δ of about 6.96–7.01 ppm), and one doublet, present at a δ of about 6.6 ppm. The signal derived from the NH group was observed as a singlet, located at a δ of about 6.9 ppm. The protons of the –CH_2_– group within the DCL moiety (C_6_H_3_C_l2_-NH-C_6_H_4_-CH_2_-COON=C-3) formed a singlet of intensity 2H, observed at a δ of 3.96–3.98 ppm. All signals confirming the presence of an oleanolate skeleton and characteristic functional groups were present in the ^1^H and ^13^C NMR spectra of the conjugates **4a**–**4d** (with a diclofenac moiety).

### 2.3. Conjugation with OAO Derivatives Increases the Cytotoxicity of Diclofenac

**Figure 2:**
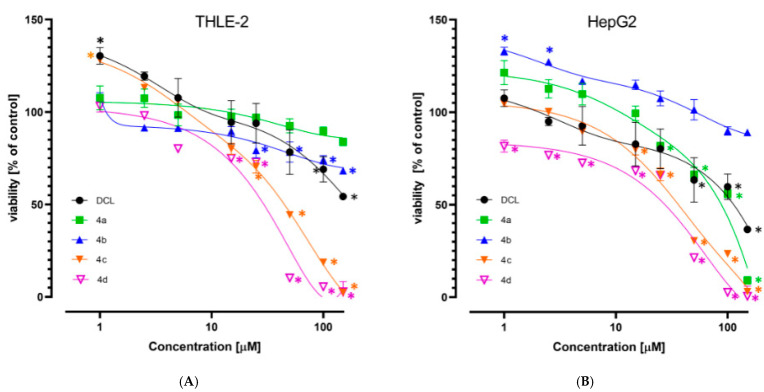
The effect of the DCL and DCL–OAO derivative conjugates (**4a**–**4d**) on the viability of THLE-2 (**A**) and HepG2 (**B**) cells (after 24 h incubation). Data (mean ± SEM) from three separate experiments are presented. * Significantly different from control—DMSO-treated cells, *p* < 0.05. Statistical significance was assessed by Student’s-*t*-test.

The viability of the immortalized hepatocytes line THLE-2, and HCC derived HepG2 cells was assessed after treatment with DCL or its conjugates at a concentration range of 1–150 µM. As shown in Figure 2A,B, DCL and conjugates (**4a**) and (**4b**) at this concentration range assured ~70% viability of both cell types, exhibiting a slightly higher cytotoxicity toward the HepG2 cells. Compounds (**4c**) and (**4d**) were more cytotoxic, as indicated by the calculated IC_50_ values (Table 1). HepG2 cells were slightly more susceptible than THLE-2 to the cytotoxic activity of the tested compounds. Based on the MTT assay results, the 10 µM and 20 µM concentrations of DCL and its conjugated OAO derivatives were applied in further assays.

### 2.4. Conjugates of Diclofenac Differently Affects the Nrf2 Activation in THLE-2 and HepG2 Cells and Expression of Nrf2 Target Genes

The activation of Nrf2 was evaluated based on the amount of Nrf2 contained in the DNA binding complex to the ARE sequence and the translocation of its active form into the nucleus. The oligonucleotides containing the ARE consensus-binding site (5′-GTCACAGTGACTCAGCAGAATCTG-3′) for Nrf2 were immobilized on microplates as bait.

**Figure 3:**
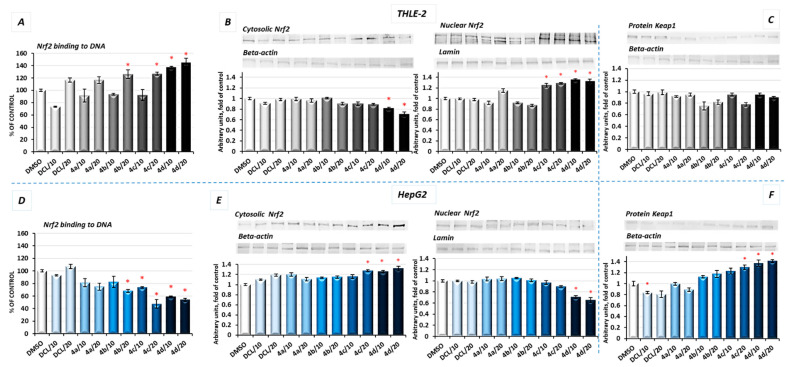
The effect of the DCL and DCL–OAO derivative conjugates (**4a**–**4d** in 10 and 20 µM concentrations) on the Nrf2–DNA binding capability, translocation from cytosol to nuclei, and Keap1 protein level in THLE-2 cells (**A**–**C**) and HepG2 cells, (**D**–**F**) after 24 h incubation. Activated Nrf2 was assessed in terms of the amount of Nrf2 contained in the DNA-binding complexes extracted from the nuclear fraction (**A**,**D**). Data (mean ± SEM) from three separate experiments in comparison to control cells set to 100%. Representative immunoblots for the analysis of the cytosolic panels and nuclear (**B**,**E**) levels of the Nrf2 protein and cytosolic Keap1 protein (**C**,**F**) are shown. The sequence of the bands corresponds to the sequence of the bars in the graph. Lamin and β-actin were used as a loading control. The values were calculated as protein levels in comparison to control cells set to 100%. * Significantly different from control—DMSO-treated cells, *p* < 0.05. Statistical significance was assessed by Student’s *t*-test.

As shown in Figure 3A, in THLE-2 cells, all conjugates except (**4a**) in a higher concentration (20 µM) increased the Nrf2 binding level. The most pronounced effect, an increase in the binding level by ~43%, was found in the case of DCL conjugated to the OAO morpholide derivative (**4d**). In HepG2 cells, a significant decrease in the Nrf2 binding level was observed as a result of treatment with methyl ester (**4b**), benzyl ester (**4c**), and morpholide (**4d**) derivatives by ~32%, ~52%, and ~46%, respectively, at the higher concentrations (Figure 3D).

The increased activation of Nrf2 in the THLE-2 cells and decrease in HepG2 cells resulting from the treatment with DCL–OAO conjugates were further confirmed by Nrf2 translocation from the cytosol to the nucleus (Figure 3B,E). Again, conjugates (**4c**) and (**4d**) were the most effective modulator of Nrf2 translocation reducing its level in the cytosol and increasing in the cell nucleus. The level of Keap1 protein decreased, but not significantly in THLE-2 cells (Figure 3C). However, in cancer cells, a ~40% increase in the cytosolic Keap1 protein level as a result of treatment with the compound (**4d**) was observed.

Activation of Nrf2 initiates the transcription of a battery of cytoprotective genes, including those encoding NAD(P)H: quinone oxidoreductase 1 (NQO1) and superoxide dismutase (SOD) [8].

**Figure 4:**
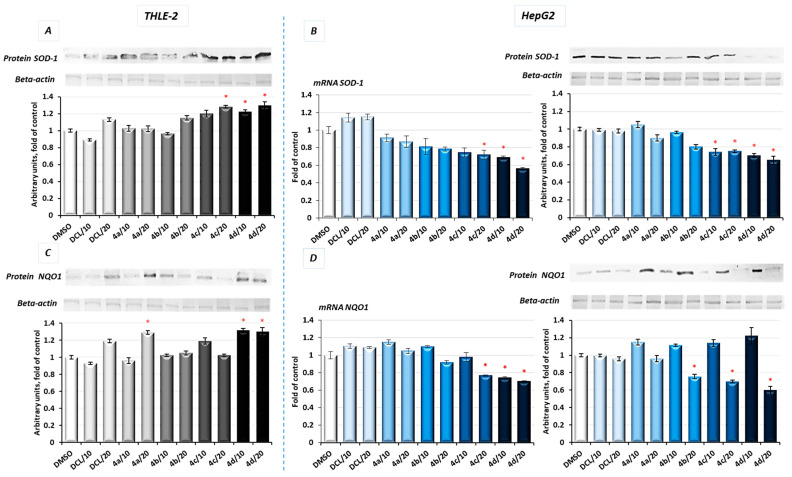
The effect of the DCL and DCL–OAO derivative conjugates (**4a**–**4d** in 10 and 20 µM concentrations) on SOD-1, (**A**,**B**) and NQO1, (**C**,**D**) mRNA and protein levels in THLE-2 and HepG2 cells after 24 h incubation. The values (mean ± SEM) for the mRNA (**B**,**D**) levels were calculated from three separate experiments in comparison to control cells set to 100%. Representative immunoblots of the cytosolic content of SOD-1 and NQO1 in THLE-2 (**A**,**C**) and HepG2 cells (**B**,**D**) from three separate experiments are shown. The sequence of the bands corresponds to the sequence of the bars in the graph. β-actin was used as a loading control. The values were calculated as protein levels in comparison to control cells set to 100%. * Significantly different from control—DMSO-treated cells, *p* < 0.05. Statistical significance was assessed by Student’s-*t*-test.

As a result of Nrf2 activation by the conjugate (**4d**), significantly increased protein levels of SOD-1 and NQO1 in immortalized hepatic normal cells were observed (Figure 4A,C).

In contrast, in the HepG2 cells, the same conjugates, i.e., (**4d**) and (**4c**), reduced the protein level of SOD-1 along with its gene transcript (Figure 4B). The NQO1 expression was significantly diminished at the mRNA level after treatment with the (**4c**) and (**4d**) derivatives. The methyl ester derivative (**4b**) also reduced the NQO1 protein level (Figure 4D). DCL did not show any significant effect on Nrf2 activation neither in THLE-2 nor HepG2 cells.

### 2.5. Molecular Docking

Molecular docking was used to elucidate whether the influence of the DCL–OAO derivative conjugates on Nrf2 activation was due to a specific interaction with the Keap-1 protein. The most active conjugates, (**4c**) and (**4d**), were the subject of the molecular docking. Additionally, the protonated version of the (**4d**) ligand was analyzed (marked as 4d_H with the protonated nitrogen atom within the morpholine system). For this purpose, the small ligand-binding C-terminal Kelch domain of the human Keap1 (PDB entry: 4XMB) was selected based on the literature data [19,20].

We focused on the global chemical reactivity descriptors, providing insights into the chemical reactivity and stability of molecules [21,22]. Therefore, we considered the neutral compounds (**4c**) and (**4d**), and from this perspective, electronegativity (χ), chemical hardness (η), first ionization potential, and electronic potential were especially interesting. The equations relating to these parameters have been described in our previous paper [23]. Computations at the B3LYP/6-311G++(d, p) level of theory proved that the mentioned descriptors were identical and equaled: χ = 3.40 eV, η = 2.40 eV, first ionization potential = 5.80 eV and electronic potential = −3.40 eV both for (**4c**) and (**4d**) derivatives. The results allowed us to assume which one of the analytes could be more potent in the chemical environment from the standpoint of their electronic nature. It was shown that (**4c**) and (**4d**) were able to interact similarly. Since the mutual ligand-amino acid contacts are crucial for determining biological activity, we concluded that particular amino acids forced these derivatives to occur in different orientations within the protein cavity during the docking protocol.

**Figure 5:**
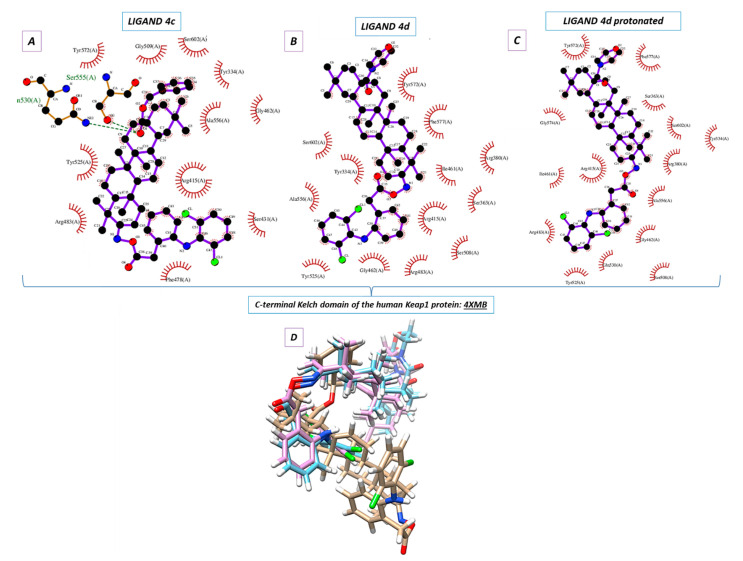
The first poses of the examined ligands ((**A**) **4c**, (**B**) **4d**, and (**C**) protonated **4d**) were determined by the Chimera 1.13.1 package. (**D**) The superimposition of the docked DCL –OAO derivative conjugates (first poses).

Nine poses were obtained for each of the DCL–OAO ligands from which the first poses had the lowest negative value for the binding affinity (Table 2). Analyzing the previously optimized ligands (Gaussian 16 C.01 program [24]) docked to the 4XMB.pdb protein, we noticed that the ligands (**4d**) and **4d_H** nearly overlapped (Figure 5B,C). The compound (**4c**) was oriented almost perpendicular to its morpholide analogs (**4d**) and **4d_H**. The resulting docking score (Table 2) pointed to the morpholide derivative (**4d**) and its protonated analog to be more potent and able to interact with the chemical environment within the cavity. Based on the literature data, the most interacting amino acid residues surrounding this cavity were selected [19,25,26].

In the docking procedure, we considered the distance *d* ≤ 4 Å between a proton and a heteroatom of the adjacent molecule (Table 3, Figure 5D) as an important factor that allows the hydrogen bonds to be formed. Thus, fitting the first poses of the ligands (**4c**), (**4d**), and **4d_H** resulted in the formation of several bonds between the DCL–OAO derivative conjugates and amino acids within the cavity of the C-terminal Kelch domain of the Keap1 protein. Apart from the (**4c**) conjugate, other derivatives accepted an H-bond from the *N*-H guanidine nitrogens of Arg415 to the nitrogen atom of the morpholide (**4d**) ligand and its protonated analog. Interaction of all analytes with Arg415 was additionally supported by contact between the chlorine atom of the DCL–OAO derivative conjugates and the N-H moiety of the arginine. Considering the distance *d* ≤ 4 Å, we observed that shorter contacts of the carbonyl group within the (**4c**) with Gln530 (2.269 Å) and Ser555 were not detected in the case of the morpholide analogs. On the contrary, the ligands (**4d**) and **4d_H** were able to interact with Arg415 (contact N^…^H-N), Gly462 (contact C-H^…^N), Gln530 (contact C-Cl^…^H-N), or Ser555 (contact C-Cl^…^H-O). Moreover, the morpholide compound’s protonation allowed forming its interaction with the hydroxyl functionality of Tyr572. Furthermore, we can assume that the Arg415 and Gln530 seem to be significant for the ligand-protein hydrogen bond formation process, and therefore for the biological response.

Based on the data from the docking protocol, ligands and residues involved in hydrogen bond formation were optimized (Sections S4 and S4.1, Supplementary Materials, Tables S2–S4) to extend the in silico studies. In Summary, Arg415 and Gln530 were crucial for hydrogen bond formation in terms of the ligand-protein complex (4XMB.pdb) interactions of the analyzed ligands in the ligand cavity (Sections S4 and S4.1, Supplementary Materials). The additional SAPT (symmetry-adapted perturbation theory) analysis of the ligand-amino acid complexes (Sections S4 and S4.2, Supplementary Materials, Table S5) was performed, and the interaction energy was estimated (Sections S4 and S4.3, Supplementary Materials, Table S6). As a result, it turned out that a more negative value of the total energy SAPT0 was obtained in the case of the derivative (**4d**) (Sections S4 and S4.2, Supplementary Materials), pointing it to be more potent for the interaction with the biological target. This conclusion was also supported by the heat of formation (HOF) computations (Mopac 2016 program and the Mozyme module [27]). The data obtained regarding the enthalpy of interactions agreed with the binding affinity estimation during the docking procedure (Sections S4 and S4.3, Supplementary Materials).

### 2.6. DCL–OAO Conjugates Diminish the Activation of NF-κB and COX-2 Expression in Normal THLE-2 Cells and Cancer HepG2 Cells

In unstimulated conditions, NF-κB is sequestered in the cytoplasm in association with its inhibitor, the IκB protein. Its activation takes place after phosphorylation of the IκB by IκB kinase beta (IKK), leading to a degradation of the inhibitory subunit, allowing free NF-κB heterodimers to migrate into the cell nucleus and to bind specific sequences of the target genes. The NF-κB generally refers to a p50-p65 heterodimer, depicting the major Rel/NF-κB complex in cells [28]. Accordingly, the effect of the DCL conjugates on NF-κB activation was evaluated based on the binding of its p50 and p65 subunits to their immobilized consensus site and the translocation of the activated complex from the cytosol into the nucleus.

**Figure 6:**
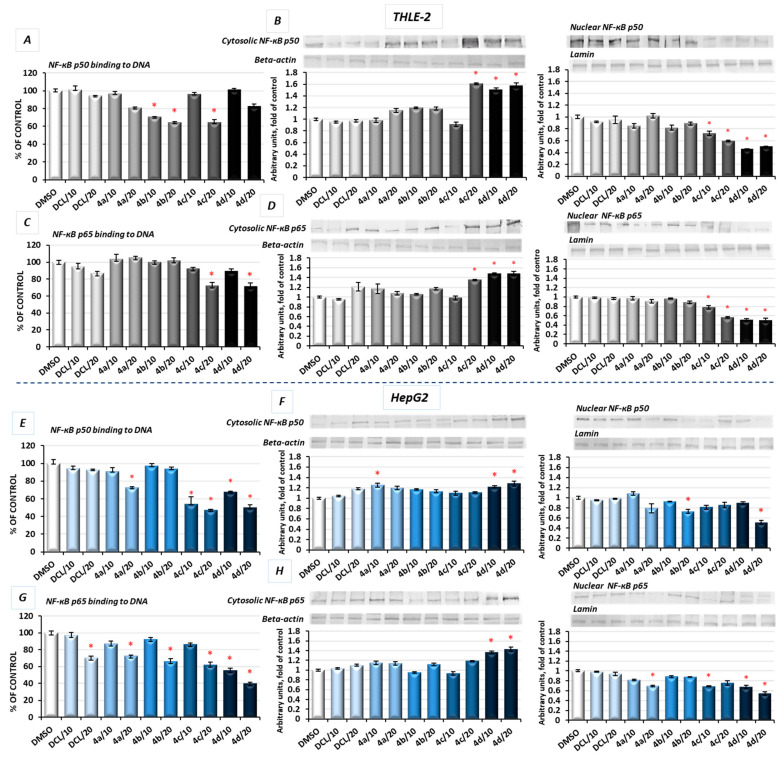
The effect of DCL and DCL–OAO derivative conjugates (**4a**–**4d** in 10 and 20 µM concentrations) on the NF-кB p50 and NF-кB p65 binding to DNA and translocation from the cytosol to the nuclei in the THLE-2 cells (**A**–**D**) and HepG2 cells (**E**–**H**) after 24 h incubation. Activated NF-кB p50 and NF-кB p65 were assessed in terms of the amount of NF-кB p50 (**A**,**E**) and NF-кB p65 (**C**,**G**) contained in the DNA-binding complexes extracted from the nuclear fraction. The values (mean ± SEM) were calculated from three separate experiments as protein levels in comparison to control cells set to 100%. Representative immunoblots for the analysis of the cytosolic and nuclear levels of proteins NF-кB p50 (**B**,**F**) and NF-кB p65 (**D**,**H**) are shown. The sequence of the bands corresponds to the sequence of the bars in the graph. Lamin and β-actin were used as a loading control. The values were calculated as protein levels in comparison to control cells set to 100%. * Significantly different from control—DMSO-treated cells, *p* < 0.05. Statistical significance was assessed by Student’s-*t*-test.

As shown in Figure 6A, in THLE-2 cells, treatment with the DCL–OAO methyl ester (**4b**) and benzyl ester (**4c**) derivative conjugates significantly diminished (by 29–36%) the content of the NF-ĸB p50 subunit in the DNA-binding complex. However, translocation of this NF-ĸB p50 subunit into the nucleus was the most affected by the DCL–OAO morpholide derivative (**4d**) (Figure 6B). Similarly, binding of the p65 subunit in concert with an increased level of its protein in the cytosol and a decrease in the nucleus was observed as a result of treatment with the DCL–OAO morpholide derivative (**4d**), and to a lesser extent the DCL–OAO benzyl derivative conjugate (**4c**) (Figure 6C,D).

DCL did not change the level of binding to DNA or translocation of both the NF-κB subunits in the THLE-2 cells.

Basically, a similar pattern was observed in the HepG2 cells (Figure 6E–H). The compounds (**4d**) and (**4c**) diminished the most significantly with binding of both NF-κB subunits to DNA. However, this effect was more pronounced in the case of the p65 subunit (up to a ~60% reduction as a result of treatment with (**4d**)) (Figure 6G). These observations were confirmed by the diminished translocation of both subunits into the nucleus (Figure 6F,H).

DCL at a concentration of 20 µM reduced the content of p65 in the DNA-binding complex but did not affect its translocation.

**Figure 7:**
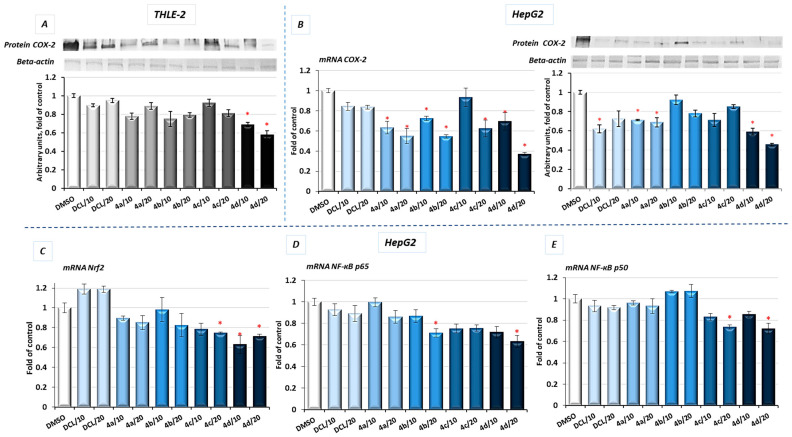
The effect of the DCL and DCL–OAO derivative conjugates (**4a**–**4d** in 10 and 20 µM concentrations) on the COX-2 protein level (**A**) in THLE-2 cells, COX-2 mRNA and protein levels in HepG2 (**B**) and the Nrf2 (**C**) and NF-κB subunits’ (**D**,**E**) transcript levels in HepG2 cells (after 24 h incubation). The values (mean ± SEM) for the mRNA levels in HepG2 cells (**B**–**E**) were calculated from three separate experiments in comparison to control cells set to 100%. Representative immunoblots of the cytosolic content of COX-2 in THLE-2 (**A**) and HepG2 cells (**B**) from three separate experiments are shown. The sequence of the bands corresponds to the sequence of the bars in the graph. β-actin was used as a loading control. The values were calculated as protein levels in comparison to control cells set to 100%. * Significantly different from control- DMSO-treated cells, *p* < 0.05. Statistical significance was assessed by Student’s-*t*-test.

*COX-2* is one of the NF-κB target genes and is often overexpressed in HCC [5]. Therefore, as a result of diminished activation of NF-κB in both tested cell lines by the compound (**4d**), a reduced level of COX-2 protein was observed. In HepG2 cells, the mRNA level of the *COX-2* was significantly decreased after treatment with all DCL–OAO derivative conjugates, similarly to its protein level, and especially after treatment with the (**4d**) conjugate (up to ~55% reduction) (Figure 7B).

These data indicate that the DCL–OAO conjugates, especially morpholide, are more efficient inhibitors of NF-ĸB in normal immortalized hepatocytes and human hepatocellular cancer cells than DCL, which, at the same concentration, showed an insignificant effect or none.

### 2.7. Conjugates of Diclofenac and OAO Derivatives Diminish the Transcription of Nrf2, NF-κB p50, and p65 in HepG2 Cells

The evaluation of the transcript levels of the Nrf2 and NF-ĸB subunits in HepG2 cells was performed to determine if the DCL–OAO conjugates may affect their expression. As shown in Figure 7, only the conjugates (**4c**) and (**4d**) reduced the Nrf2 and NF-ĸB p50 mRNA levels, while (**4b**) and (**4d**) significantly decreased the p65 mRNA level. DCL alone tended to increase Nrf2 transcript levels and decrease both NF-ĸB subunits, but the observed differences in comparison with untreated control cells were not statistically significant. These results indicate that DCL–OAO conjugates, particularly with the benzyl and morpholide groups at the C-17 position, not only diminished the activation of key signaling pathways involved in HCC development but also affected the expression of the transcription factors participating in the final stages of these pathways.

### 2.8. Bead-Based Multiplex Immunoassay Revealed Possible Downregulation of Protein Regulating Several Signaling Pathways

**Figure 8:**
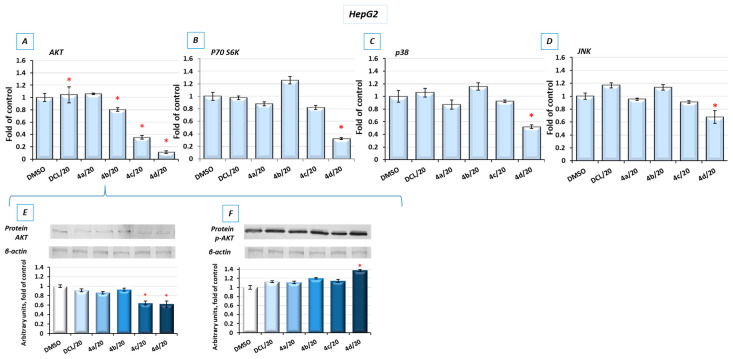
The effect of the DCL and DCL–OAO derivative conjugates (**4a**–**4d** in 20 µM concentration) on the cytosolic level of AKT (**A**), p70 S6 kinase (**B**), p38 (**C**), and JNK (**D**) measured in the cytosolic fractions of the HepG2 cells (after 24 h incubation) and calculated on the MAGPIX^®^ System. (**E**,**F**) The protein levels of AKT and p-AKT (phosphorylation site Ser 473), respectively assessed by a Western blot, where β-actin was used as a loading control. The results expressing the mean fluorescence intensity (MFI) are presented as the fold of control from three independent measurements. The values (mean ± SEM) were calculated as protein levels in comparison to control cells set to 100%. * Significantly different from control, DMSO-treated cells, *p* < 0.05. Statistical significance was assessed by Student’s-*t*-test.

To look into possible interferences of the tested conjugates with the other signaling pathways involved in cell survival, the bead-based multiplex immunoassay was applied. As shown in Figure 8, conjugates (**4d**) and, to a lesser extent, (**4c**) reduced the level of kinases from the MAPK family, p38 and JNK, AKT, and P70S6K involved in multiple cellular processes, including apoptosis, cell proliferation, and protein synthesis [29], respectively. For verification, the results of the bead-based multiplex immunoassay, and a Western blot analysis of AKT and p-AKT were performed (Figure 8E,F). The reduction in AKT levels was also noticeable, although the observed changes were not as significant as with the use of the bead-based immunoassay. The level of phosphorylated AKT (p-AKT) in this assay was slightly increased.

### 2.9. Conjugates of Diclofenac and OAO Derivatives Change the Cell Cycle Distribution, Induce Apoptosis and Affect Cell Proliferation in HepG2 Cells

**Figure 9:**
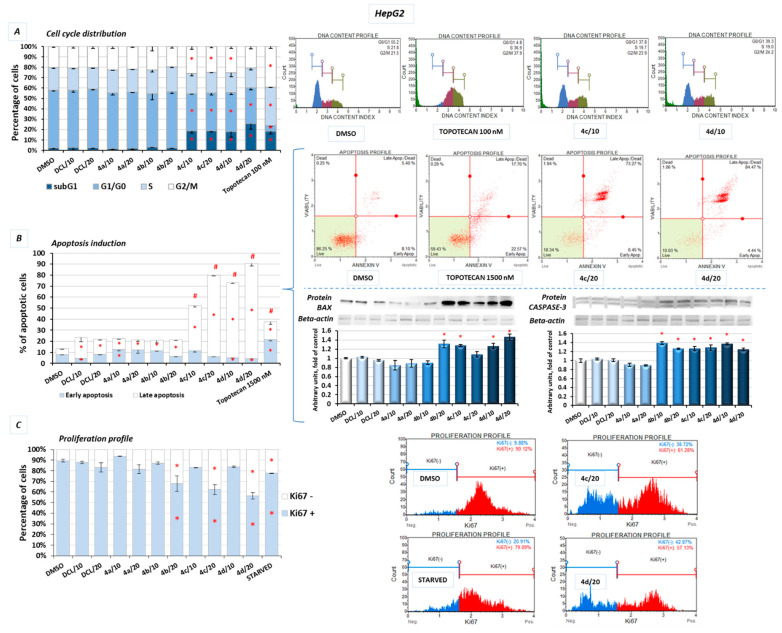
The effect of the DCL and DCL–OAO derivative conjugates (**4a**–**4d** in 10 and 20 µM concentrations) on the cell cycle distribution (**A**), apoptosis induction (**B**), and on the proliferation level (**C**) in HepG2 cells (after 24 h incubation). (**A**) Graphs represent the percentage of cells in the subG1 (green color before the G1 phase on plots), G1/G0, S, and G2/M phases measured by flow cytometry after propidium iodide staining. Topotecan was used as a positive control. (**B**) Graphs represent the percentage of cells in the early and late stages of the apoptosis assessed by flow cytometry measurements based on the signal from Annexin V bound to phosphatidylserine externalized in apoptotic cells. A dead cell marker, 7-AAD, was also included. Topotecan was used as a reference for proapoptotic activity. The protein levels of Bax and Caspase-3 is also presented. (**C**) Graphs representing the percentage of proliferating (Ki67(+)) and nonproliferating (Ki67(−)) cells measured by flow cytometry. Starved cells (cultured in an FBS-free medium) were used as a reference for the anti-proliferative conditions. Representative plots and immunoblots are presented. β-actin was used as a loading control for protein analysis. For all panels, the mean values ± SEM from three independent experiments is shown. Asterisks (*) denote statistically significant changes from control—DMSO treated cells, and panel B denotes statistically significant changes in the percentage of either early or late apoptotic cells, while hashes (#) denote statistically significant changes in the percentage of total apoptotic cells, *p* < 0.05. Statistical significance was assessed by Student’s *t*-test.

Figure 9A shows the effect of the DCL–OAO derivatives on the cell cycle distribution in the HepG2 cells. Conjugates of DCL with OAO derivatives substituted with benzyl (**4c**) and morpholide (**4d**) groups increased the number of cells significantly in the G2/M and subG1 phases. They also decreased the number of cells in the G0/G1 phases (Figure 9A). However, compared to the reference compound, topotecan, which at low concentrations induces cell cycle arrest in S and G2/M phases, the effect of the DCL–OAO derivative conjugates was much weaker. DCL, similarly to compounds (**4a**) and (**4b**), did not affect the cell cycle distribution.

Treatment with the DCL–OAO derivatives (**4a**) and (**4b**) increased the percentage of early and late apoptotic cells to an extent comparable to DCL. However, in the case of the (**4c**) and (**4d**) conjugates, the proapoptotic effect exceeded even that observed after treatment with topotecan. The highest percentage of total apoptotic cells after treatment with (**4c**) and (**4d**) was marked by ~67% and ~77%, respectively, compared to the results from the DMSO-treated cells (Figure 9B).

The pro-apoptotic effect of these compounds was further confirmed by an increased level of the Bax and Caspase-3 proteins.

The effect of the DCL conjugates on cell proliferation was evaluated based on the Ki67 protein expression, allowing the assessment of the cell’s proliferation rate. The most pronounced anti-proliferative effect among the tested derivatives was observed, similarly as in the previous assays, as a result of treatment with the compounds (**4c**) and (**4d**). In this regard, the highest percentage of resting cells (43.5%) (exhibiting the lowest Ki67 expression) was found after treatment with the (**4d**) derivative at its higher concentration. The percentage of resting cells treated with these compounds exceeded the amount observed in the starved cells lacking FBS in DMEM (Figure 9C).

Reduced activation of both transcription factors, NF-κB, which may enhance anti-apoptotic gene expression, and Nrf2 controlling genes whose products are involved in cell proliferation and differentiation may be responsible for the cell cycle arrest and induction of apoptosis by the DCL–OAO derivative conjugates.

### 2.10. Conjugates of Diclofenac and OAO Derivatives Affect the Phosphorylation of ERK and Formation of ROS in HepG2 Cells

**Figure 10:**
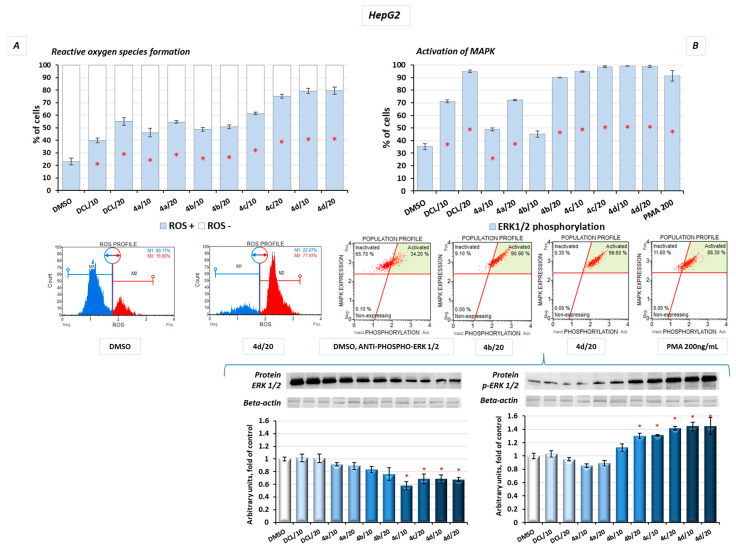
The effect of the DCL and DCL–OAO derivative conjugates (**4a**–**4d** in 10 and 20 µM concentrations) on the generation of reactive oxygen species (ROS (+)) (**A**) and induction of ERK phosphorylation (**B**) in the HepG2 cells (after 24 h incubation). (**A**) Graphs representing the relative percentage of cells that are ROS negative and positive (percentage of cells undergoing oxidative stress based on the intracellular detection of superoxide radicals). The kit based on dihydroethidium was used to detect ROS-exhibiting cells (ROS (+)). (**B**) Graphs representing the percentage of cells expressing ERK 1/2 protein in an active (phosphorylated) state. Phorbol myristate acetate (PMA)-stimulated cells were used as a positive control. The protein levels of ERK 1/2 and p-ERK 1/2 was also shown. Representative plots and immunoblots are presented. β-actin was used as a loading control for protein analysis. The ERK 1/2 antibody used recognizes only its non-phosphorylated form. For all panels, the mean values ± SEM from three independent experiments are shown. Asterisks (*) denote statistically significant changes from control—DMSO-treated cells, *p* < 0.05. Statistical significance was assessed by Student’s *t*-test.

Additionally, in the HepG2 cells, after treatment with the DCL–OAO derivative conjugates, the levels of reactive oxygen species (ROS) were assessed. Induction of oxidative stress was dose-dependent, with the most significant increase in ROS levels resulting from treatment with the derivatives (**4c**) and (**4d**) at a concentration of 20 µM, by ~57% and ~60%, respectively, compared to the DMSO-treated cells (Figure 10A).

The mitogen-activated protein kinases (MAPK) family, including extracellular signal-regulated kinases (ERK), is known to be directly activated by the ROS and regulates the growth and survival of cancer cells. MAPK pathways are frequently deregulated in human hepatocarcinogenesis. Increased ERK phosphorylation relating to ROS production was observed as a result of treatment with the conjugates tested in this study. The results confirmed that prolonged ERK activation requires the presence of the ROS, and almost the whole population of the HepG2 cells presented MAPK signaling activation (increased phosphorylation of ERK 1/2) in flow cytometric analysis after treatment with the DCL–OAO derivatives with benzyl ester and morpholide at both tested concentrations (Figure 10B). A similar increase was obtained in the protein levels, where we observed a ~30% and ~40% increase of p-ERK after treatment with the conjugates (**4c**) and (**4d**), respectively (Figure 10B).

### 2.11. Antitumor Efficacy of DCL–OAO Derivatives in Human Hepatic Tumor Xenograft Growth

**Figure 11:**
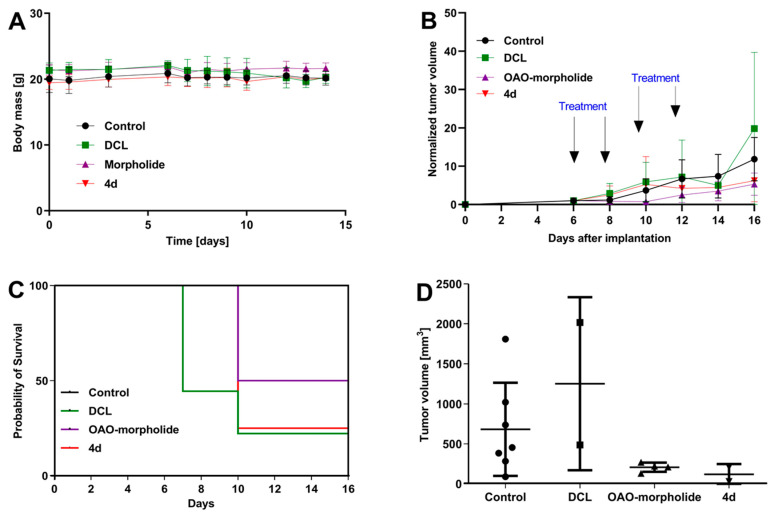
Anti-cancer efficacy of the DCL, and OAO morpholide derivative and their conjugation in HepG2 tumor xenografts. (**A**) The body weight of animals during the experiment. (**B**) The growth curves of the HepG2 tumor xenografts. (**C**) Kaplan-Meier curves for animals bearing the HepG2 xenografts. (**D**) Tumor volumes measured ex vivo following the sacrifice of the mice. The volumes of the tumors on day 6, when tumors were measurable in all animals, were set as 1.

We evaluated the anti-cancer efficacy of the DCL–OAO derivative conjugates in nude mice bearing HepG2 tumor xenografts. As shown in Figure 11, DCL did not significantly affect the tumor growth compared to the untreated mice. At sacrifice, tumor volumes were significantly reduced by the OAO morpholide derivative and to a lesser extent by its conjugation to DCL. No significant difference in mice weights at sacrifice was observed between the different treatment groups.

**Figure 12:**
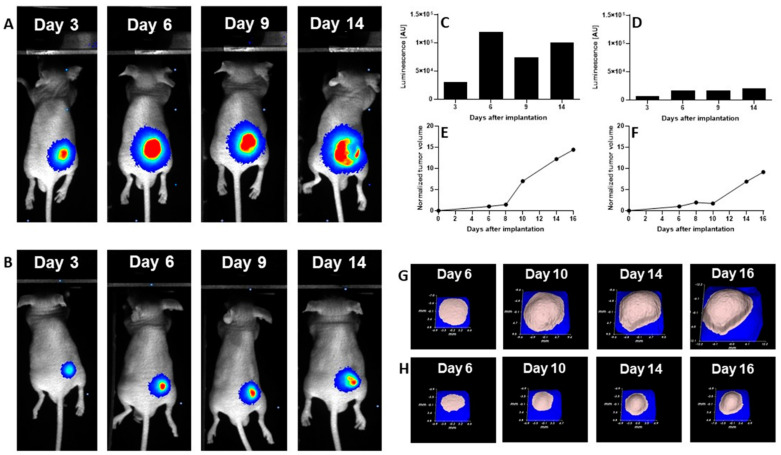
(**A**) The luminescence images of a representative control mouse, while (**B**) shows the luminescence images of a representative mouse receiving OAO–morpholide. (**C**,**D**) Luminescence measured in regions of interest (ROI) in mice receiving: vehicle and OAO–morpholide. (**E**,**F**) Tumor volume changes (expressed as a normalized tumor volume) in representative control and OAO–morpholide-treated mice, respectively, while (**G**,**H)** shows corresponding pictures obtained using the Peira Instrument (ultrasound-based) (**G**) from control mouse, and (**H**) from a mouse receiving OAO–morpholide.

It should be noted that Figure 11 shows the number of animals at the end of the experiment. At the beginning, each group included eight mice. Unfortunately, the treatment with two tested compounds (OAO morpholide and **4d**) was toxic for the animals. The tendency to develop signs of necrosis in certain xenografts impeded the measurement of the luminescence signal. However, the obtained data confirmed the results of the biometric measurements (Figure 12).

## 3. Discussion

Diclofenac, a widely used NSAID, has shown several biological activities which might be useful in cancer therapy or prophylaxis [13]. Moreover, recent studies showed that in combination with other drugs such as sorafenib, diclofenac displayed greater antiproliferative efficacy in HCC cells and anti-tumor activity in vivo in comparison to DCL or sorafenib alone [15]. Our study focused on the evaluation of synthetic hybrids of DCL with OAO derivatives in the context of modifications to signaling pathways critical for cell survival. The results showed that the conjugation of DCL, particularly with OAO substituted with morpholide in C-17 (**4d**), significantly increased toxicity toward both cancer HepG2 and immortalized non-tumor THLE-2 cells. However, in the THLE-2 cells, this effect was slightly less pronounced.

Indeed, as demonstrated in our earlier study, OAO derivatives, particularly OAO- morpholide, showed cytotoxic effects in HepG2 cells and to a lesser extent in immortalized normal hepatocyte THLE-2 cells [10].

It is worth noting the marked differences between the ASP, IND, and DCL conjugates on the parameters evaluated in the hepatic cells. Therefore, for DCL, similarly, as for the other NSAIDs studied so far, conjugation with the OAO derivatives is certainly an advantage (e.g., decreased IC_50_ values) and might improve the DCL therapeutic potential.

It should be pointed out that at the lowest concentrations an increased cell proliferation was observed indicating a hormetic effect [30]. Such an effect might be related to a dysfunction in redox homeostasis and an initial compensation reaction in the presence of the lowest concentration of compounds, particularly those with antioxidant potential [31].

One of the reasons leading to cell death may be an excessive generation of ROS. Indeed, in HepG2 cells, the ROS levels were significantly increased as a result of treatment with all tested compounds, but the highest percentage of ROS-positive cells (>80%) was found in the case of the most cytotoxic—(**4c**) and (**4d**)—conjugates.

The results of an earlier analysis of the transcriptome in primary human hepatocytes revealed that a majority of intrinsic hepatotoxic drugs activated the Nrf2 transcriptional program. Furthermore, strong Nrf2 activation corresponded with the downregulation of genes under the direct control of NF-κB [32]. This mechanism may explain the cytotoxicity of the DCL–OAO derivative hybrids (**4c**) and (**4d**) toward the THLE-2 cells, in which increased activation of Nrf2 and subsequently increased expression of its target genes was observed with concomitant reduced activation of NF-κB. This cell line represents the immortalized human liver epithelial (non-tumor) cells characterized by an overall higher metabolic activity in comparison to the HepG2 cells. Besides, although they have normal hepatic epithelial cell morphology, their features make them a good model of liver tumor promotion and chemoprevention [33]. The activation of Nrf2 in these cells as a result of treatment with potential chemopreventive agents such as betanin was found in our earlier studies [34].

HepG2 cells are derived from hepatocellular carcinomas and represent the later stages of hepatocarcinogenesis. In these cells, the conjugates (**4c**) and (**4d**) decreased the Nrf2 activation in contrast to the OAO-derivatives alone observed in our earlier studies [35]. Several mechanisms may be involved in Nrf2 activation and inhibition.

Probst et al. [36] demonstrated that pharmacological Keap1 inhibition by the synthetic triterpenoid RTA 408, is distinct from those resulting from the loss of functional Keap1 in tumor cells and does not increase cellular proliferation or survival.

The results of the molecular docking performed in this study demonstrated that the compound (**4d**) is able to bind to Keap1 and this suggests that in normal cells, Nrf2 activation may occur through the stabilization of Keap1. In cancer cells, increased ERK phosphorylation relating to ROS production was observed as a result of treatment with the DCL–OAO derivative conjugates, indicating the contribution of this mechanism to their cytotoxicity. On the other hand, the relationship between ERK and Nrf2 activation was described [37]. While in the case of the OAO derivatives alone, both mechanisms, i.e., the down-regulation of Keap1 and activation of ERK, might contribute to Nrf2 activation and cytotoxicity, their conjugation with DCL or indomethacin [11] results in the opposite effect. Therefore, the mechanism of Nrf2 inhibition by the DCL–OAO derivative hybrids in HCC cells requires further study. Nrf2 activation leads to the transcription of several genes, including expression of the *SOD*-1 and *NQO*1 genes, whose products are important for protection against ROS. SOD-1 catalyzes the dismutation of the superoxide-free radical anion into molecular oxygen and hydrogen peroxide, the substrate of catalase, and/or glutathione peroxidase [38]. Similarly, *NQO*1, besides its catalytic action in reducing quinones, may also directly scavenge superoxide [39]. Therefore, reduced expression of both *SOD*-1 and *NQO1* in HepG2 cells may contribute to the increased ROS levels in these cells and ultimately cell death.

Sustained Nrf2 activity in cancer cells may promote cancer growth and increase chemoresistance [7]. Thus, the reduced activation of Nrf2 along with an increased generation of ROS in cancer cells is highly desirable, and the compounds (**4c**) and (**4d**) seem to act in this way. It is worth noting that DCL did not show any significant effects on Nrf2 activation in neither the THLE-2 nor HepG2 cells.

The activation of NF-κB measured in terms of binding of its active subunits p65 and p50 into DNA, and their translocation from the cytosol into the nucleus was reduced as an effect from treatment with the DCL–OAO morpholide derivative (**4d**) and to a lesser extent the conjugate with the OAO benzyl ester (**4c**). The p65 subunit is basically responsible for the transcription initiation, and its binding to DNA was more affected. The p50 subunit serves only as a helper in NF-κB DNA binding [40,41].

The disproportionate increase in the active p65 subunits and ultimately of effector molecules is fundamental to the pathogenesis of many diseases, including cancer. Henceforth, the NF-κB p65 signaling pathway is recognized as an important target for novel drug design, discovery, and development [38]. Notably, DCL-conjugates decreased the level of *COX*-2 protein in both lines but most significantly in the HepG2 cells. Since an overexpression of *COX*-2 is related to increased cell growth and is observed in human HCC, the most potent conjugate of DCL (**4d**) might be considered one of the candidates for new therapeutics. Again, DCL alone did not affect the NF-κB activation in THLE-2 and only slightly in the HepG2 cells, indicating that conjugation to the OAO derivatives may enhance its anti-cancer activity.

To explain the mechanism of cell death induced by these compounds, their effect on cell cycle distribution and apoptosis was assessed. While cell cycle distribution was not affected, apoptosis was induced by all conjugates, but the proapoptotic effect of compounds (**4c**) and (**4d**) exceeded induction of that of topotecan used as a reference compound. Reduced activation of both transcription factors, NF-κB, which may enhance proapoptotic gene expression, and Nrf2 controlling genes whose products are involved in cell proliferation and differentiation, along with reduced activation of kinases from the MAPK family, may also be responsible for the induction of apoptosis by the DCL–OAO derivative conjugates. Inhibition of these signaling pathways by the compounds (**4c**) and (**4d**) also resulted in reduced proliferation of the HepG2 cells.

Importantly, the anti-cancer activity of the DCL–OAO derivatives was confirmed in vivo. While DCL had little effect on tumor volume, its conjugate with the OAO morpholide decreased, although to a lesser extent than this derivative alone. The relatively high mortality level observed after treatment with these compounds was observed in mice bearing HepG2 tumor xenografts.

One reason which might explain this effect not occurring in normal mice is their metabolism to more toxic derivatives. In support of this concept in several tumors, a temporary decrease in the luminescence signal during the experiment was observed while the tumor volume was continuously increasing. This effect may be related to the disordered distribution of luciferin in the tumor due to blood vessel disruption or necrotic core formation. Further studies are required to explain the mechanism of antitumor activity of the DCL and OAO–morpholide conjugates in vivo. The data obtained in this experiment will be useful in designing a new protocol for HepG2 cell implantation as well as for compound delivery and dosing regimen, which should fully demonstrate their therapeutic potential.

In summary, this study showed that the conjugation of DCL with OAO derivatives, particularly OAO–morpholide, enhanced DCL anti-cancer activity in HCC. Reduced activation of Nrf2 and NF-κB along with other signaling molecules leading to apoptosis and limitations to cell proliferation may be responsible for these effects. In normal immortalized cells, through activation of Nrf2, the same conjugate may exert a chemopreventive effect. Although the results of the tumor xenografts experiment are promising, further studies are needed to confirm in vitro observations.

## 4. Materials and Methods

### 4.1. Chemistry

#### 4.1.1. Synthesis and Purification of the DCL–OAO Derivative Conjugates

General information concerning the performed chemical experiments and the elucidation of the synthesized compounds’ chemical structures are presented in our earlier publication [10]. The purity of the obtained compounds was evaluated based on spectra data: IR, NMR, EI-MS, and elemental analysis (Supplementary Materials, Sections S2 and S3, Table S1, Figures S1–S8).

#### 4.1.2. Computational Details

The optimization of all ligands using the Gaussian 16 C.01 program [24], Mopac 2016 software [27], and interaction enthalpy calculations (basing on the heat of formation values) were carried out based on our previous publication [42]. The QM calculations were carried out using resources provided by the Wroclaw Center for Networking and Supercomputing (*Bem* cluster). The human Keap1 Kelch domain in complex with a 2,2′-(naphthalene-1,4-diylbis(((4-methoxyphenyl)sulfonyl)azanediyl))diacetamide, acquired from the Protein Data Bank base (PDB entry: 4XMB.pdb with a resolution of 2.43 Å), was selected as the biological target [19] as one of the most used docking PDB versions of the human Keap1 Kelch protein. To carry out docking simulation (using the AutoDock Vina package [43]), a grid box was defined to be of 10 Å size (center _x = 4.096, center = −0.355, center = −22.022). The outputs (*.*pdbqt* files) after the docking procedure were visualized using the Chimera 1.13.1 package [44]. The projections of the 1st poses of ligands docked to the protein were visualized with the LigPlot+ v.2.2 software [45,46].

### 4.2. Biological Assays

#### 4.2.1. Cell Culture and Viability Assay

HepG2 (ATCC HB 8065) and THLE-2 (ATCC CRL-2706) cells were provided by the American Type Culture Collection (ATCC, Manassas, VA, USA). HepG2 cells were maintained in Dulbecco’s Modified Eagle’s Medium (DMEM, Sigma-Aldrich, St. Louis, MI, USA) containing 10% fetal bovine serum (FBS, EURx, Gdansk, Poland) and 1% of antibiotic solution (Sigma-Aldrich, St. Louis, MI, USA), while THLE-2 were cultured in BEGM supplemented with the Bullet Kit (Lonza, Cologne, Germany) and 10% FBS, 5 ng/mL EGF, 70 ng/mL phosphoethanolamine at 37 °C, in a humidified, 5% CO_2_ atmosphere. To assess the effect of the DCL and DCL–OAO conjugates on measured parameters, 1 × 10^6^ cells were seeded per 100 mm of the culture dish. After 24 h of initial incubation, the cells were treated with 10 µM and 20 µM of DCL and its conjugates **4a**–**4d** and 0.1% of dimethyl sulfoxide (DMSO) control solution. Incubation lasted for 24 h, and the cells were then harvested.

The effect of tested compounds on cell viability was assessed by the MTT assay, following the standard protocol. Briefly, THLE-2 and HepG2 cells were seeded (10^4^ per well) in 96-well plates. After 24 h of preincubation in a complete medium, compounds were added in various concentrations, and cells were incubated for 24 h. Later, cells were washed twice with phosphate-buffered saline (PBS) and further incubated for 4 h with a medium containing 0.5 mg/mL 3-(4,5-dimethylthiazol-2-yl)-2,5-diphenyl-2H-tetrazolium bromide (MTT). Then, the formazan crystals were dissolved in acidic isopropanol, and the absorbance was measured at 570 nm and 690 nm. All experiments were repeated three times. In all following experiments, we used non-toxic concentrations of compounds (with viability level above 70%): 10 and 20 μM of DCL and its OAO conjugates (**4a**–**4d**). The molar masses and corresponding concentrations of compounds expressed in g/mol and µg/mL, respectively, are presented in Table 4.

#### 4.2.2. Nuclear and Cytosolic Fractions Preparation

According to the manufacturer’s protocol, the subcellular extracts from HepG2 and THLE-2 cells were prepared using the Nuclear/Cytosol Fractionation Kit (BioVision Research, Milpitas, CA, USA). Protein concentration was assessed, and the samples were stored at −80 °C for further analysis.

#### 4.2.3. Total RNA Isolation, cDNA Synthesis, and Quantitative Real-Time PCR (RT-PCR)

The extraction of total RNA was performed by the GeneMatrix Universal DNA/RNA/Protein Purification Kit (EURx, Gdańsk, Poland). Subsequently, samples were subjected to reverse transcription by the RevertAid First Strand cDNA Synthesis Kit (Thermo Fisher Scientific, Waltham, MA, USA), according to the manufacturer’s instructions.

For the quantitative R-T PCR analysis, the Maxima SYBR Green Kit (Fermentas Inc., USA) and the BioRad Chromo4 thermal cycler (BioRad Laboratories, Hercules, CA, USA) were used. The protocol started with 5 min of enzyme activation at 95 °C, followed by 40 cycles of 95 °C for 15 s, 56 °C for 20 s and 72 °C for 40 s, and a final elongation at 72 °C for 5 min. Melting curve analysis was used for amplicon verification. The expression of *TBP* (*TATA box binding protein*) and *PBGD* (*porphobilinogen deaminase*) was used to normalize the data. The Pfaffl comparative method was used for fold-change quantification. Primers were designed using the Beacon Designer software and subjected to a BLAST search to minimize unspecific binding. Only the primer pairs that generated intron-spanning amplicons were selected. The primer sequences used to analyze *Nrf2*, *NF-кBp65*, *NF-кBp50*, *SOD-1*, *NQO1*, *COX-2*, *TBP*, and *PBGD* genes are listed in Table 5.

#### 4.2.4. Western Blot Analysis

Cytosolic extracts of Nrf2, NF-кB p65, NF-кB p50, SOD-1, NQO1, COX-2, Keap1, AKT, p-AKT, Bax, Caspase-3, ERK 1/2 and p-ERK 1/2, and β-actin, or nuclear extracts for Nrf2, NF-кB p65, NF-кB p50, and lamin protein detection, were separated on 12% or 10% SDS-PAGE slab gels. Β-actin and lamin were used as a loading control. The amount of cytosolic and nuclear fractions was equal to 100 µg of protein per well. Proteins were transferred to the nitrocellulose Immobilon P membrane. After blocking for 2 h with 10% skimmed milk, proteins were probed with rabbit anti-Nrf2 (sc-13032), rabbit anti-NF-кB p65 (sc-7151), rabbit anti-NF-кB p50 (sc-114), rabbit anti-SOD-1 (sc-8637), goat anti-NQO1 (sc-16464), rabbit anti-COX-2 (sc-376861), goat anti-Keap1 (sc-15246), goat anti-AKT (sc-1619), rabbit anti-p-AKT (phosphorylation site Ser 473) (sc-101629), mouse anti-Bax (sc-493), mouse anti-Caspase-3 (sc-271028), mouse anti-ERK ½(sc-135900) and goat anti-p-ERK ½ (phosphorylation site Thr 202 and Tyr 204) (sc-16982), rabbit anti-β-actin (sc-sc-7210), and rabbit anti-lamin (sc-206800) (Santa Cruz Biotechnology, Dallas, TX, USA). Alkaline phosphatase AP-labeled anti-rabbit IgG, anti-goat IgG, anti-mouse IgG secondary antibodies (BioRad Laboratories, Hercules, CA, USA), and the horseradish peroxidase HRP-conjugated anti-rabbit IgG, anti-goat IgG, anti-mouse IgG secondary antibodies (Bosterbio, Pleasanton, CA, USA) were used in the staining reaction. Bands were visualized using the AP Conjugate Substrate Kit NBT/BCIP, or the chemiluminescent HRP Substrate Clarity ECL Kit (BioRad Laboratories, Hercules, CA, USA). The amount of immunoreactive products in each lane was determined using the ChemiDoc Imaging System (BioRad Laboratories, Hercules, CA, USA). Values were calculated as relative absorbance units (RQ) per mg of protein and expressed as a percentage of the control.

#### 4.2.5. Nrf2 and NF-ĸB Binding Assay

Nrf2, NF-κB p50, and NF-κB p65 activation were assessed by the enzymatic immunoassay (Transcription Factor ELISA Assay Kit Active Motif, Belgium) according to the manufacturer’s instructions. Activated Nrf2 was evaluated based on the amount of Nrf2 contained in the DNA-binding complex to the ARE sequence. The oligonucleotides containing the ARE consensus-binding site (5′-GTCACAGTGACTCAGCAGAATCTG-3′) for Nrf2 were immobilized on microplates as bait. Activated NF-ĸB was measured as the amount of p65 and p50 subunits held in the DNA-binding complex. The oligonucleotides containing (5’-GGGACTTTCC-3′), a consensus site for NF-κB, were immobilized on microplates as bait. Nuclear fractions were incubated with oligonucleotides for 1 h, wells were washed, and DNA-bound subunits were detected by a specific primary antibody and a secondary antibody conjugated with HRP. The results were expressed as the normalized level of absorbance (OD450 nm per mg of protein).

#### 4.2.6. Cell Cycle Distribution

The analysis of the cell cycle distribution was performed using the Muse^®^ Cell Cycle Kit (Merck, Darmstadt, Germany) according to the manufacturer’s protocol. Briefly, cells (3 × 10^5^ per well) were seeded and grown in 6-well plates for 24 h before stimulation. Then, the tested compounds were added, and cells were incubated for a further 24 h. Topotecan (100 nM)-treated cells were used as a positive control for the cell cycle arrest. Subsequently, cells were harvested by trypsinization, washed with PBS buffer, fixed in ice-cold 70% ethanol, and frozen at −20 °C until staining. After overnight storage, cells were washed with cold PBS buffer, stained with propidium iodide in the presence of RNAase A, and after a 30 min incubation in the dark at room temperature the fluorescence signal was analyzed by flow cytometry on the Muse^®^ Cell Analyzer. Data were evaluated using the Muse^®^ 1.5 Analysis Software.

#### 4.2.7. Apoptosis

The externalization of phosphatidylserine is a marker of apoptotic processing and was analyzed by the Annexin V staining using the Muse^®^ Annexin V & Dead Cell Kit (Merck, Darmstadt, Germany) according to the manufacturer’s protocol. Discrimination between early- and late-apoptotic cells was reached by 7-Aminoactinomycin D (7-AAD) staining. Briefly, 3 × 10^5^ cells per well were seeded and grown in 6-well plates for 24 h before stimulation. Then, the tested compounds were added, and cells were incubated for a further 24 h. Topotecan (1500 nM)-treated cells were used as a positive control for apoptosis induction. Subsequently, cells were collected by trypsinization and stained with Annexin V and 7-AAD solution. After a 20 min incubation in the dark at room temperature, the cells were analyzed by flow cytometry on the Muse^®^ Cell Analyzer, and data were evaluated using the Muse^®^1.5 Analysis software.

#### 4.2.8. Proliferation

The Ki67 protein was used as a marker for proliferating cells, and its expression was detected by the fluorochrome-labeled antibody from the Muse^®^ Ki67 Proliferation Kit (Merck, Darmstadt, Germany) according to the manufacturer’s protocol. Briefly, cells (3 × 10^5^ per well) were seeded in 6-well plates. After 24 h, the tested compounds were added, and cells were incubated for a further 24 h. Cells cultured in a medium without FBS (starved cells) were used as a positive control for antiproliferative conditions. After the incubation, cells were collected by trypsinization, washed with PBS buffer, instantly fixed, and exposed to a permeabilization buffer. After a 30 min incubation in the dark at room temperature with the Muse^®^ Hu Ki67-PE antibody, cells were analyzed by flow cytometry on the Muse^®^ Cell Analyzer, and data were evaluated using the Muse^®^1.5 Analysis Software.

#### 4.2.9. ROS Generation

Dihydroethidium (DHE) reacts with superoxide radicals, generating fluorophores with a high affinity to DNA. The Muse^®^ Oxidative Stress Kit (Merck, Darmstadt, Germany) containing DHE was used according to the manufacturer’s protocol for the quantitative measurement of the superoxide radicals’ positive cells. Briefly, cells (3 × 10^5^ per well) were seeded in 6-well plates. After 24 h, the tested compounds were added, and cells were incubated for a further 24 h. Subsequently, cells were collected by trypsinization, washed with PBS buffer, resuspended in 1X Assay Buffer, mixed with Muse^®^ Oxidative Stress Reagent working solution, and subjected to a 30 min incubation (37 °C, 95% humidity, 5% CO_2_). Cells were analyzed by flow cytometry on the Muse^®^ Cell Analyzer, and data were evaluated using the Muse^®^ 1.5 Analysis Software.

#### 4.2.10. ERK1/2 Phosphorylation Assay

ERK1/2 phosphorylation is the marker of the active state of the canonical MAPK signaling pathway. The Muse^®^ MAPK Activation Dual Detection Kit (Merck, Darmstadt, Germany) was used according to the manufacturer’s protocol. Briefly, cells (3 × 10^5^ per well) were seeded in 6-well plates. After 24 h preincubation, the tested compounds were added, and the cells were grown for an additional 24 h. PMA (200 ng/mL)-treated cells were used as a positive control for the MAPK pathway activation. Subsequently, cells were collected by trypsinization, washed with PBS buffer, fixed, exposed to a permeabilization buffer, and incubated for 30 min in the dark at room temperature with a mixture of two directly conjugated antibodies: anti-phospho-ERK1/2 (Thr202/Tyr204, Thr185/Tyr187)-PE antibody (detection of cells expressing phosphorylated ERK1/2) and an anti-ERK1/2-PECy5 conjugated antibody (detecting all cells expressing ERK1/2). Cells were analyzed by flow cytometry on the Muse^®^ Cell Analyzer, and data were evaluated using the Muse^®^ 1.5 Analysis Software.

#### 4.2.11. Bead-Based Immunoassay on the Luminex MAGPIX Instrument

Magnetic bead-based immunoassay was performed on the Luminex-MAGPIX multiplex immunoassay system. Data were analyzed with the Milliplex Analyst 5.1 software (EMD Millipore, Burlington, MA, USA). The panel we performed included quantitation of the following proteins in the cytosolic fractions of HepG2 cells, AKT, p70 S6, p38, and JNK, according to the manufacturer’s instructions. The magnetic bead panel with high sensitivity antibodies were obtained from Merck, Germany. A multiplex test based on microspheres using the Luminex^®^ xMAP^®^ technology with different fluorescent colors was detected on a compatible MAGPIX^®^ camera. Cytosolic fractions were suspended in MILLIPLEX^®^ MAP buffer. The bead suspension was added to each well of a 96-well plate, and samples were added into the wells and incubated overnight at 2–8 °C on a shaker protected from light. The plate was washed with 2X buffer, and then 1X MILLIPLEX^®^ MAP Detection Antibodies were added. After shaking for 1 hour at room temperature, the antibodies were removed, and 1X MILLIPLEX^®^ MAP Streptavidin/Phycoerythrin (SAPE) was added. Then, the MILLIPLEX^®^ MAP Amplification Buffer was added to each well and shaken for 15 min. The beads were suspended in MILLIPLEX^®^ MAP buffer, and each microsphere was identified with a MAGPIX^®^ Luminex Analyzer, and the results were calculated from the reporter’s fluorescent signals. Mean fluorescence intensities were quantified with the xPonent 4.2 software (Luminex Corporation, Austin, TX, USA). The raw MFI results for the tested protein levels were converted relative to the control of DMSO-treated cells.

#### 4.2.12. In Vivo Tumor Growth

Animal experiments were performed with the approval from the Poznan Local Ethics Committee for the Animal Experiments (date of approval: 5 April 2019; decision number 11/2019). Eight-week-old male nude athymic BALB/c nu/nu mice were purchased from the Charles River Laboratory (Sulzfeld, Germany). The animals were housed in individually ventilated cages at a 12/12 h light/dark cycle and provided ad libitum with standard diet and water.

HepG2 cells were stably transfected with a luciferase reporter gene using the following protocol Vector: pGL4.51 (Promega, Madison, WI, USA), transfection reagent: Viafect (Promega, Madison, WI, USA) ratio DNA: transfection reagent- 1:4, incubation time with vector: 48 h. For selection: G418 at a dose of 5000 µg/mL was used, and the cells were maintained in selection medium for two weeks in DMEM (+10% FBS, −P/S). The obtained clones were checked in the Ivis Spectrum (Caliper Life Sciences, Waltham, MA, USA) system using luciferin (GoldBio, St. Louis, MI, USA) (30 mg/mL) in PBS.

HepG2 cells were collected from the flasks, counted and resuspended at a concentration of 8 × 10^6^ per 100 µL of cold PBS and implanted subcutaneously into the right flank. The implantation of the xenograft was checked after five days. For this purpose, the luciferin (GoldBio, St. Louis, MI, USA) (150 mg/mL) was injected intraperitoneally. The animals were transferred from the anesthesia chamber to the Photon Imager instrument (BioSpace Lab, Nesles-la-Vallée, France) where they were kept under isoflurane-induced anesthesia. Then, the luminescence was monitored in the selected region of interest until a plateau had been reached.

Once tumor xenografts reached ~50 mm^3^, mice were randomized into four treatment groups (*n* = 8/group; control, DCL, the OAO–morpholide derivative, and its DCL conjugate (**4d**)).

Mice were treated intraperitoneally twice a week for 14 days (total of 4 applications) with 20 mg/kg b.w. of DCL, the OAO–morpholide derivative, and its DCL conjugate (**4d**) dissolved in ethanol: PEG400:0.9% NaCl at proportion 1:2:1. The control group received 50 µL of vehicle alone. Changes in tumor volume were recorded daily using the contactless measuring device Peira TM900 (Peira, Belgium). The tumor growth was also monitored using a bioimaging method. For this purpose, the luciferin (150 mg/mL) was injected intraperitoneally. The animals were transferred from the anesthesia chamber, and then the luminescence was monitored until a plateau had been reached. The obtained results were analyzed using the M3vision software. All surviving animals were sacrificed after 14 days of monitoring due to the excessive tumor burden.

### 4.3. Statistical Analysis

Statistical analysis was performed using the GraphPad program (GraphPad Software, San Diego, CA, USA), assuming a significance level of changes at *p* < 0.05. The student’s *t*-test assessed the statistical significance between the experimental groups and the control.

## 5. Conclusions

Our study demonstrated that the conjugation of diclofenac with the novel OAO morpholide and benzyl ester enhanced its anti-cancer activity in HCC. The reduced activation of Nrf2 and NF-κB leading to the induction of apoptosis and reduced cell proliferation may be responsible for this effect. In non-tumor cells, activation of Nrf2 by these compounds provides the basis for chemopreventive strategies by inhibiting carcinogenesis initiation and promotion. Further studies are required to explain the mechanism of the DCL–OAO derivative conjugates’ anti-cancer activity in vivo.

## Figures and Tables

**Table 1:** Comparison of the DCL and DCL–OAO derivatives cytotoxicity in THLE-2 and HepG2 cells *.

Compound	THLE-2	HepG2
DCL	>150	122 ± 6.88
4a	>150	107 ± 3.81
4b	>150	>150
4c	44.5 ± 3.46	37 ± 0.52
4d	34 ± 5.07	33.5 ± 1.83

* IC_50_ values ± SEM [µM].

**Table 2:** Estimated binding affinity [kcal/mol] for the first two poses of the DCL–OAO derivative conjugates generated during the docking procedure; **4d_H**—protonated form of the (**4d**) derivative.

Pose	Estimated Binding Affinity		
	4c	4d	4d_H
1	−9.800	−10.900	−10.600
2	−9.200	−10.600	−10.500

**Table 3:** Ligand-amino acid contacts (under *d* ≤ 4.0 Å) for the first poses of the DCL–OAO derivative conjugates generated during the docking procedure.

Contact	Contacts Calculated for Docked DCL–OAO Derivatives		
	4c	4d	4d_H
N^…^H-N_Arg415_	×	2.992	2.894
C-Cl^…^H-N_Arg415_	3.474	2.938	3.516
C-H^…^N(H)_Gly462_	×	3.352	3.026
C=O^…^H-N_Gln530_	2.269	×	×
C-Cl^…^H-N_Gln530_	×	3.607	3.573
C=O^…^H-O_Ser555_	2.501	×	×
C-Cl^…^H-O_Ser555_	×	2.688	2.737
N-H^…^O_Tyr572_	×	×	2.648

**Table 4:** The molar masses and corresponding concentrations of compounds expressed in g/mol and µg/mL, respectively.

Compound	The Molar Mass [g/mol]	C_mol_ = 10 µM [Converted to µg/mL]	C_mol_ = 20 µM [Converted to µg/mL]
Sodium DCL	318.13	3.1813	6.3626
4a	747.85	7.4785	14.9570
4b	761.87	7.6187	15.2374
4c	837.97	8.3797	16.7594
4d	816.95	8.1695	16.3390

**Table 5:** Primers used in R-T PCR.

Gene	Forward Primer	Reverse Primer
*NRF2*	5′ATTGCTACTAATCAGGCTCAG	5′GTTTGGCTTCTGGACTTGG
*SOD-1*	5′CGACAGAAGGAAAGTAATG	5′TGGATAGAGGATTAAAGTGAGG
*NQO1*	5′CAATTCAGAGTGGCATTC	5′GAAGTTTAGGTCAAAGAGG
*NF-ĸB p50*	5′ATCATCCACCTTCATTCTCAA	5′AATCCTCCACCACATCTTCC
*NF-ĸB p65*	5′CGCCTGTCCTTTCTCATC	5′ACCTCAATGTCCTCTTTCTG
*COX-2*	5′CCTGTGCCTGATGATTGC	5′CAGCCCGTTGGTGAAAGC
*PBGD*	5′TCAGATAGCATACAAGAGACC	5′TGGAATGTTACGAGCAGTG
*TBP*	5′GGCACCACTCCACTGTATC	5′GGGATTATATTCGGCGTTTCG

## Data Availability

Data is contained within the article and Supplementary Materials.

## Supplementary Materials

The following are available online at https://www.mdpi.com/article/10.3390/ph14070688/s1, **Content:** **1.** An overview of the synthesis of the novel DCL–OAO derivative conjugates (**4a**–**4d**) (Scheme S1). **2.** Detailed spectral characteristics of the novel DCL–OAO derivative conjugates (**4a**–**4d**) (Table S1). **3**. ^1^H NMR and ^13^C NMR spectra of the novel DCL–OAO derivative conjugates (**4a**–**4d**) (Figures S1–S8). **4.** Expanded description of the in silico studies on ligands **4c** and **4d** interactions with the Kelch binding domain of the Keap1 protein. **4.1.** Optimization of ligands and residues involved in hydrogen bond formation. **4.2.** The SAPT analysis of ligand-amino acid complexes. **4.3.** Estimation of the interaction energy. Figure S1. The ^1^H NMR for compound **4a**, Figure S2. The ^13^C NMR for compound **4a**, Figure S3. The ^1^H NMR for compound **4b**, Figure S4. The ^13^C NMR for compound **4b**, Figure S5. The ^1^H NMR for compound **4c**, Figure S6. The ^13^C NMR for compound **4c**, Figure S7. The ^1^H NMR for compound **4d**, Figure S8. The ^13^C NMR for compound **4d**. Table S2. Ligand amino acid contacts (under *d* ≤ 4.0 Å) for the first poses of DCL-OAO derivatives conjugates after ligands optimization with B3LYP/6-31G(d,p) method (ONIOM method, model 1); RMSDcomplex = 0.430 (**4c**), 0.372 (**4d**), 0.460 (**4d_H**), Å, respectively. Table S3. Ligand amino acid contacts (under *d* ≤ 4.0 Å) for the first poses of DCL-OAO derivatives conjugates after ligands optimization with PM6 method (ONIOM method, model 2); RMSDcomplex = 0.240 (**4c**), 0.499 (**4d**), 0.515 (**4d_H**), Å, respectively. Table S4. Ligand amino acid contacts (under *d* ≤ 4.0 Å) for the first poses of OAO–DCL derivatives conjugates after ligands optimization with PM7 method (ONIOM method, model 3); RMSDcomplex = 0.364 (**4c**), 0.351 (**4d**), 0.535 (**4d_H**), Å, respectively. Table S5. Calculated total values of the interaction ligand amino acid energy [kcal/mol] using the SAPT0 method for docked DCL-OAO derivatives conjugates. Table S6. Ligand amino acid contacts (under *d* ≤ 4.0 Å) for the first poses of DCL-OAO derivatives conjugates after ligands optimization with PM7 method; RMSDcomplex = 0.893 (**4c**), 1.652 (**4d**), 1.244 (**4d_H**), Å, respectively.
