# Supplementary material for: Conjugation of Diclofenac with Novel Oleanolic Acid Derivatives Modulate Nrf2 and NF-κB Activity in Hepatic Cancer Cells and Normal Hepatocytes Leading to Enhancement of Its Therapeutic and Chemopreventive Potential
Source: Pharmaceuticals (Basel). 2021 Jul 17;14(7):688. doi: 10.3390/ph14070688 (PMC8308582; doi:10.3390/ph14070688)
Supplement: Supplementary file 1 [file pharmaceuticals-14-00688-s001.zip › pharmaceuticals-1244560-supplementary.pdf]

<u>Supplementary Materials</u>

CONJUGATION OF DICLOFENAC WITH NOVEL OLEANOLIC ACID DERIVATIVES MODULATE NRF2 AND NF-KB ACTIVITY IN HEPATIC CANCER CELLS AND NORMAL HEPATOCYTES LEADING TO ENHANCEMENT OF ITS THERAPEUTIC AND CHEMOPREVENTIVE POTENTIAL

Maria Narożna[1], Violetta Krajka-Kuźniak[1], Barbara Bednarczyk-Cwynar[2], Małgorzata Kucińska[3], Robert Kleszcz[1], Jacek Kujawski[2], Hanna Piotrowska-Kempisty[3], Adam Plewiński[4], Marek Murias[3], Wanda Baer-Dubowska[1*]

[1] Department of Pharmaceutical Biochemistry, Poznan University of Medical Sciences, 60-781 Poznan, Poland

[2] Department of Organic Chemistry, Poznan University of Medical Sciences, 60-780 Poznan, Poland

[3] Department of Toxicology, Poznan University of Medical Sciences, 60-631 Poznan, Poland

[4] Centre for Advanced Technologies, Adam Mickiewicz University, 61-614, Poznan, Poland

*Corresponding author
e-mail: baerw@ump.edu.pl, phone: (+48) 61 854 66 25, fax: (+48) 61 854 66 20

<u>Content:</u>

1. An overview of the synthesis of the novel DCL-OAO (**4a** - **4d**) (**Scheme S1**).

2. Detailed spectral characteristics of the novel DCL-OAO (**4a** - **4d**) (**Table S1**).

3. ^{1}H NMR and ^{13}C NMR spectra of the novel DCL-OAO (**4a** - **4d**) (**Figures S1 – S8**).

4. Expanded description of the *in silico* studies on ligands **4c** and **4d** interactions with the Kelch binding domain of the Keap1 protein.

4.1. *Optimization of ligands and residues involved in hydrogen bond formation.*

4.2. *The SAPT analysis of ligand-amino acid complexes.*

4.3. *Estimation of the interaction energy.*

1. Scheme S1. An overview of the synthesis of the novel DCL-OAO derivatives (**4a** - **4d**).

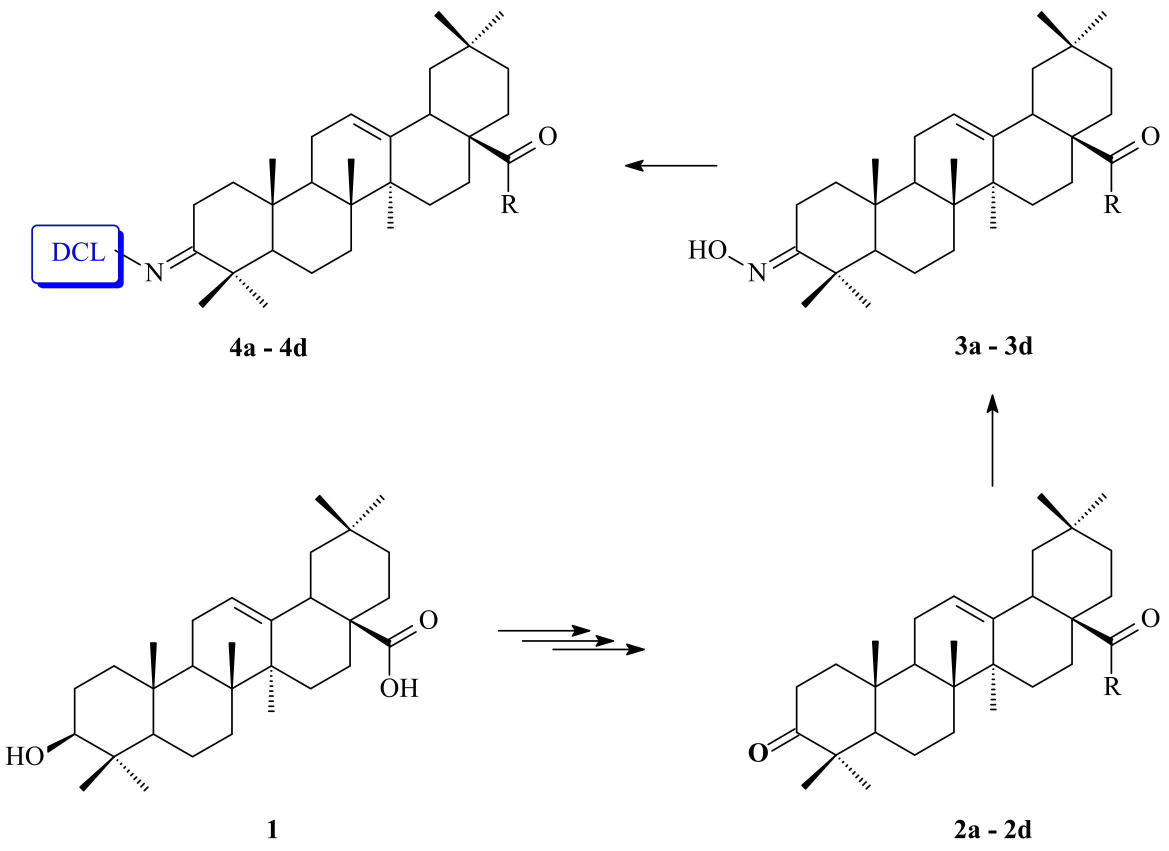

2a, 3a, 4a: R = H
2b, 3b, 4b: R = OCH_3
2c, 3c, 4c: R = CH_2-Ar
2d, 3d, 4d: R = morpholine ring

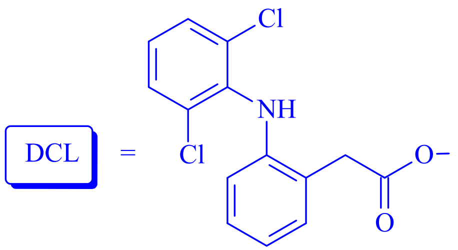

2. Table S1. Detailed spectral characteristics of the novel DCL-OAO (**4a** - **4d**).

		Spectral information
**4a**	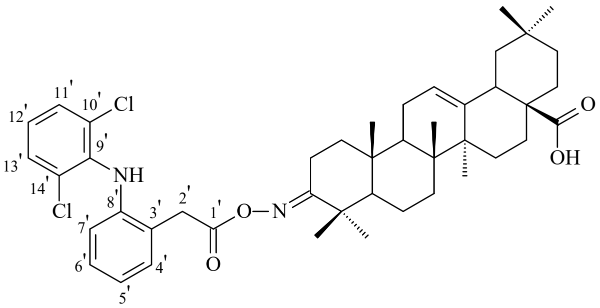	**4a**<br>*3-diclofenacoxyiminoolean-12-en-28-oic acid*<br>**$C_{44}H_{56}Cl_2N_2O_4$.**<br>**mol. mass**: 746.362.<br>**yield**: 693 mg (92.8 %).<br>**m.p.**: 100 – 103 ^{o}C, amorphous powder.<br>**IR** (KBr, cm^{-1}) ν: 3380 (N-H, $C_6H_3Cl_2$-**NH**-C_6H_4-CH_2-COON=C-3); 3335 (CH, $\mathbf{C_6H_3}Cl_2$-NH-$\mathbf{C_6H_4}$-CH_2-COON=C-3); 1735 (C=O, $C_6H_3Cl_2$-NH-C_6H_4-CH_2-**CO**ON=C-3); 1725 (N=C, $C_6H_3Cl_2$-NH-C_6H_4-CH_2-COO**N=C-**3); 1700 (C=O, -**CO**OH).<br>**^{13}C NMR** (151 MHz, $CDCl_3$) δ: 183.65 (C_q, -**CO**OH, C-28); 175.89 (C_q, $C_6H_3Cl_2$-NH-C_6H_4-CH_2-**CO**ON=C-3, C-1′); 170.55 (C_q, C-3); 143.83 (C_q, C-13); 142.84 x 2 (C_q x 2, $\mathbf{C_6H_3}Cl_2$-NH-C_6H_4-CH_2-COON=C-3, C-10′ and C-14′); 137.95 (C_q, $\mathbf{C_6H_3}Cl_2$-NH-C_6H_4-CH_2-COON=C-3, C-9′); 130.87 x 2 (CH x 2, $\mathbf{C_6H_3}Cl_2$-NH-C_6H_4-CH_2-COON=C-3, C-11′ and C-13′); 129.51 (CH, $C_6H_3Cl_2$-NH-$\mathbf{C_6H_4}$-CH_2-COON=C-3, C-6′); 128.83 (CH, $C_6H_3Cl_2$-NH-$\mathbf{C_6H_4}$-CH_2-COON=C-3, C-7′); 127.96 (CH, $C_6H_3Cl_2$-NH-$\mathbf{C_6H_4}$-CH_2-COON=C-3, C-4′); 127.94 (C_q, $C_6H_3Cl_2$-NH-$\mathbf{C_6H_4}$-CH_2-COON=C-3, C-8′); 124.43 (C_q, $C_6H_3Cl_2$-NH-$\mathbf{C_6H_4}$-CH_2-COON=C-3, C-3′); 123.89 (CH, $\mathbf{C_6H_3}Cl_2$-NH-C_6H_4-CH_2-COON=C-3, C-12′); 122.41 (CH, C-12); 118.23 (CH, $C_6H_3Cl_2$-NH-$\mathbf{C_6H_4}$-CH_2-COON=C-3, C-5′); 60.41 (CH_2, $C_6H_3Cl_2$-NH-C_6H_4-$\mathbf{CH_2}$-COON=C-3, C-2′); 46.77 (C_q, C-17).<br>**^{1}H NMR** (600 MHz, $CDCl_3$) δ: 7.35 and 7.34 (2 x 1H, 2 x s, $\mathbf{C_6H_3}Cl_2$-NH-C_6H_4-CH_2-COON=C-3, C-11′-H and C-13′-H), 7.27 (1H, dd, *J* = 7.5 and 1.1 Hz, $C_6H_3Cl_2$-NH-$\mathbf{C_6H_4}$-CH_2-COON=C-3, C-6′-H); 7.13 (1H, td, *J* = 7.6 and 1.3 Hz, $\mathbf{C_6H_3}Cl_2$-NH-C_6H_4-CH_2-COON=C-3, C-12′-

		H); 7.00 (1H, t, *J* = 8.1 Hz, $C_6H_3Cl_2$-NH-**C_6H_4**-CH_2-COON=C-3, C-5′H); 6.97 (1H, t, *J* = 7.3 Hz, $C_6H_3Cl_2$-NH-**C_6H_4**-CH_2-COON=C-3, C-4′-H); 6.88 (1H, s, $C_6H_3Cl_2$-**NH**-C_6H_4-CH_2-COON=C-3); 6.58 (1H, d, *J* = 8.0 Hz, $C_6H_3Cl_2$-NH-**C_6H_4**-CH_2-COON=C-3, C-7′-H); 5.29 (1H, t, *J* = 3.4 Hz, C-12-H); 3.98 (2H, s, $C_6H_3Cl_2$-NH-C_6H_4-**CH_2**-COON=C-3, C-2′-H_2); 2.84 (1H, dd, *J* = 13.8 and 4.1 Hz, C-18-H_β); 1.14, 0.94, 0.93, 0.91, 0.87, 0.86, 0.75 (7 x 3H, 7 x s, 7 x CH_3 groups).<br>**MS/EI** (m/z): 747.84 (12.4%, $M^{\bullet+}$).<br>**Elem. anal.**: for $C_{44}H_{56}Cl_2N_2O_4$ calcd.: C: 70.67%, H: 7.55%, N: 3.75%, found: C: 70.59%, H: 7.58%, N: 3.76%.
**4b**	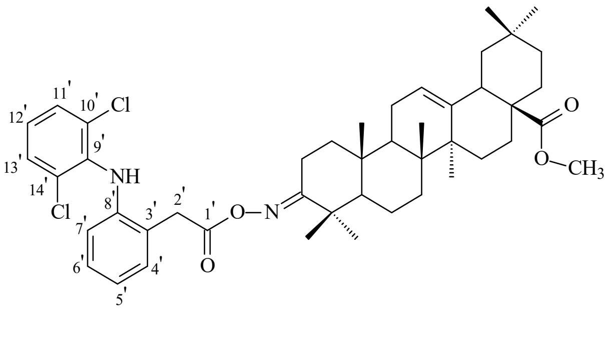	**4b**<br>***<u>3-diclofenacoxyiminoolean-12-en-28-oic acid methyl ester</u>***<br>**$C_{45}H_{58}Cl_2N_2O_4$**.<br>**mol. mass**: 760.377.<br>**yield**: 730 mg (96.1 %).<br>**m.p.**: 195 – 196 °C, needles (EtOH).<br>**IR** (KBr, cm^{-1}) ν: 3385 (N-H, $C_6H_3Cl_2$-**NH**-C_6H_4-CH_2-COON=C-3); 3330 (CH, **C_6H_3**Cl_2-NH-**C_6H_4**-CH_2-COON=C-3); 1735 (C=O, $C_6H_3Cl_2$-NH-C_6H_4-CH_2-**CO**ON=C-3); 1725 (N=C, $C_6H_3Cl_2$-NH-C_6H_4-CH_2-COO**N=C**-3); 1720 (C=O, -**CO**OCH_3).<br>**^{13}C NMR** (151 MHz, $CDCl_3$) δ: 178.24 (C_q, -**CO**OCH_3, C-28); 175.83 (C_q, $C_6H_3Cl_2$-NH-C_6H_4-CH_2-**CO**ON=C-3, C-1′); 170.53 (C_q, C-3); 143.94 (C_q, C-13); 142.89 x 2 (C_q x 2, **C_6H_3**Cl_2-NH-C_6H_4-CH_2-COON=C-3, C-10′ and C-14′); 137.93 (C_q, **C_6H_3**Cl_2-NH-C_6H_4-CH_2-COON=C-3, C-9′); 130.91 x 2 (CH x 2, **C_6H_3**Cl_2-NH-C_6H_4-CH_2-COON=C-3, C-11′ and C-13′); 129.54 (CH, $C_6H_3Cl_2$-NH-**C_6H_4**-CH_2-COON=C-3, C-6′); 128.82 (CH, $C_6H_3Cl_2$-NH-**C_6H_4**-CH_2-COON=C-3, C-7′); 127.97 (CH + C_q, $C_6H_3Cl_2$-NH-**C_6H_4**-CH_2-COON=C-3, C-4′ and C-8′); 124.32 (C_q, $C_6H_3Cl_2$-NH-**C_6H_4**-

		CH_2-COON=C-$_3$, C-3′); 123.98 (CH, **C_6H_3**Cl_2-NH-C_6H_4-CH_2-COON=C-$_3$, C-12′); 122.05 (CH, C-12); 118.44 (CH, $C_6H_3Cl_2$-NH-**C_6H_4**-CH_2-COON=C-$_3$, C-5′); 60.40 (CH_2, $C_6H_3Cl_2$-NH-C_6H_4-**CH_2**-COON=C-$_3$, C-2′); 51.56 (CH_3, -COO**CH_3**); 45.83 (C_q, C-17).<br>**^{1}H NMR** (600 MHz, $CDCl_3$) δ: 7.35 and 7.34 (2 x 1H, 2 x s, **C_6H_3**Cl_2-NH-C_6H_4-CH_2-COON=C-$_3$, C-$_{11'}$-H and C-$_{13'}$-H); 7.27 (1H, dd, *J* = 7.5 and 1.1 Hz, $C_6H_3Cl_2$-NH-**C_6H_4**-CH_2-COON=C-$_3$, C-$_{6'}$-H); 7.13 (1H, td, *J* = 7.6 and 1.3 Hz, **C_6H_3**Cl_2-NH-C_6H_4-CH_2-COON=C-$_3$, C-$_{12'}$-H); 6.99 (1H, t, *J* = 6.1 Hz, $C_6H_3Cl_2$-NH-**C_6H_4**-CH_2-COON=C-$_3$, C-$_{5'}$-H); 6.98 (1H, t, *J* = 7.3 Hz, $C_6H_3Cl_2$-NH-**C_6H_4**-CH_2-COON=C-$_3$, C-$_{4'}$-H); 6.89 (1H, s, $C_6H_3Cl_2$-**NH**-C_6H_4-CH_2-COON=C-$_3$); 6.57 (1H, d, *J* = 8.0 Hz, $C_6H_3Cl_2$-NH-**C_6H_4**-CH_2-COON=C-$_3$, C-$_{7'}$-H); 5.31 (1H, t, *J* = 3.4 Hz, C_{12}-H); 3.97 (2H, s, $C_6H_3Cl_2$-NH-C_6H_4-**CH_2**-COON=C-$_3$, C-$_{2'}$-H_2); 3.65 (3H, s, -COO**CH_3**); 2.89 (1H, dd, *J* = 13.8 and 4.1 Hz, C-$_{18}$-H_β); 1.29, 1.16, 1.14, 1.04, 0.95, 0.92, 0.78 (7 x 3H, 7 x s, 7 x CH_3 groups).<br>**MS/EI** (m/z): 760.77 (16.6%, $M^{\bullet+}$).<br>**Elem. anal.**: for $C_{45}H_{58}Cl_2N_2O_4$ calcd.: C: 70.94%, H: 7.67%, N: 3.68%, found: C: 71.03%, H: 7.51%, N: 3.60%.
**4c**	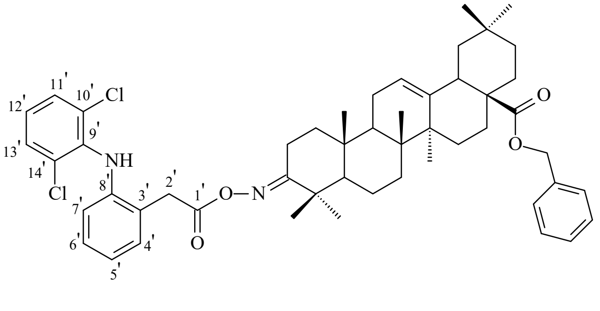	**4c**<br>***<u>3-diclofenacoxyiminoolean-12-en-28-oic acid benzyl ester</u>***<br>**$C_{51}H_{62}Cl_2N_2O_4$.**<br>**mol. mass**: 836.409.<br>**yield**: 786 mg (94.0 %).<br>**m.p.**: 65 – 70 ^{o}C, amorphous powder.<br>**IR** (KBr, cm^{-1}) ν: 3385 (N-H, $C_6H_3Cl_2$-**NH**-C_6H_4-CH_2-COON=C-$_3$); 3325 (CH, **C_6H_3**Cl_2-NH-**C_6H_4**-CH_2-COON=C-$_3$); 2900 (CH, -COOCH_2**C_6H_5**); 1740 (C=O, $C_6H_3Cl_2$-NH-C_6H_4-CH_2-

		**CO**ON=C-$_3$); 1720 (N=C, $C_6H_3Cl_2$-NH-C_6H_4-CH_2-COO**N=C**-$_3$); 1705 (C=O, -**CO**O$CH_2C_6H_5$).<br>**^{13}C NMR** (151 MHz, $CDCl_3$) δ: 177.39 (C_q, **-CO**O$CH_2C_6H_5$, C-28); 175.84 (C_q, $C_6H_3Cl_2$-NH-C_6H_4-CH_2-**CO**ON=C-$_3$, C-1′); 170.54 (C_q, C-3); 143.83 (C_q, C-13); 142.90 x 2 (C_q x 2, **C_6H_3**Cl_2-NH-C_6H_4-CH_2-COON=C-$_3$, C-10′ and C-14′); 137.94 (C_q, **C_6H_3**Cl_2-NH-C_6H_4-CH_2-COON=C-$_3$, C-9′); 136.41 (C_q, -COO-CH_2-**C_6H_5**); 130.92 and 130.87 (CH x 2, **C_6H_3**Cl_2-NH-C_6H_4-CH_2-COON=C-$_3$, C-11′ and C-13′); 129.56 (CH, $C_6H_3Cl_2$-NH-**C_6H_4**-CH_2-COON=C-$_3$, C-6′); 128.83 (CH, $C_6H_3Cl_2$-NH-**C_6H_4**-CH_2-COON=C-$_3$, C-7′); 128.43 x 2, 128.03, 128.02 and 127.95 (CH x 5, **-**COOCH_2**C_6H_5**); 127.99 (CH, $C_6H_3Cl_2$-NH-**C_6H_4**-CH_2-COON=C-$_3$, C-4′); 127.94 (C_q, $C_6H_3Cl_2$-NH-**C_6H_4**-CH_2-COON=C-$_3$, C-8′); 124.31 (C_q, $C_6H_3Cl_2$-NH-**C_6H_4**-CH_2-COON=C-$_3$, C-3′); 124.00 (CH, **C_6H_3**Cl_2-NH-C_6H_4-CH_2-COON=C-$_3$, C-12′); 122.21 (CH, C-12); 118.43 (CH, $C_6H_3Cl_2$-NH-**C_6H_4**-CH_2-COON=C-$_3$, C-5′); 65.96 (CH_2, -COO**CH_2**C_6H_5); 60.41 ($C_6H_3Cl_2$-NH-C_6H_4-**CH_2**-COON=C-$_3$, C-2′); 46.76 (C_q, C-17).<br>**^{1}H NMR** (600 MHz, $CDCl_3$) δ: 7.41 – 7.31 (7H, m, -COO-CH_2-**C_6H_5** and **C_6H_3**Cl_2-NH-C_6H_4-CH_2-COON=C-$_3$, C-$_{11'}$-H and C-$_{13'}$-H); 7.26 (1H, dd, *J* = 7.5 and 1.1 Hz, $C_6H_3Cl_2$-NH-**C_6H_4**-CH_2-COON=C-$_3$, C-$_{6'}$-H); 7.14 (1H, td, *J* = 7.7 and 1.9 Hz, **C_6H_3**Cl_2-NH-C_6H_4-CH_2-COON=C-$_3$, C-$_{12'}$-H); 6.99 (1H, t, *J* = 8.1 Hz, $C_6H_3Cl_2$-NH-**C_6H_4**-CH_2-COON=C-$_3$, C-$_{5'}$-H); 6.96 (1H, t, *J* = 7.3 Hz, $C_6H_3Cl_2$-NH-**C_6H_4**-CH_2-COON=C-$_3$, C-$_{4'}$-H); 6.90 (1H, s, $C_6H_3Cl_2$-**NH**-C_6H_4-CH_2-COON=C-$_3$); 6.58 (1H, d, *J* = 8.0 Hz, $C_6H_3Cl_2$-NH-**C_6H_4**-CH_2-COON=C-$_3$, C-$_{7'}$-H); 5.33 (1H, t, *J* = 3.5 Hz, C_{12}-H); 5.11 (2H, d, *J* = 12.6 Hz, -COO**CH_2**C_6H_5); 3.97 (2H, s, $C_6H_3Cl_2$-NH-C_6H_4-**CH_2**-COON=C-$_3$, C-$_{2'}$-H_2); 2.95 (1H, dd, *J* = 4.0 and 13.7 Hz, C-$_{18}$-$H_β$); 1.30, 1.16, 1.14; 1.02; 0.95, 0.93; 0.66 (7 x 3H, 7 x s, 7 x CH_3).<br>**MS/EI** (m/z): 837.04 (11.1%, $M^{•+}$).

		**Elem. anal.**: for $C_{51}H_{62}Cl_2N_2O_4$ calcd.: C: 73.10%, H: 7.46%, N: 3.34%, found: C: 73.03%, H: 7.50, %, N: 3.36%.
**4d**	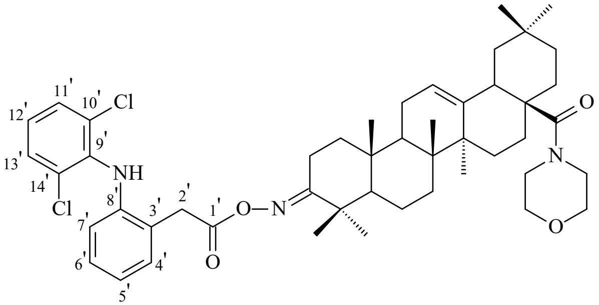	**4d**<br>***<u>3-diclofenacoxyiminoolean-12-en-28-oic acid morpholide</u>***<br>**$C_{48}H_{63}Cl_2N_3O_4$.**<br>**mol. mass**: 815.420.<br>**yield**: 770 mg (94.4 %).<br>**m.p.**: 77 – 82 °C, amorphous powder.<br>**IR** (KBr, cm^{-1}) ν: 3380 (N-H, $C_6H_3Cl_2$-**NH**-C_6H_4-CH_2-COON=C-3); 3335 (CH, **C_6H_3**Cl_2-NH-**C_6H_4**-CH_2-COON=C-3); 1735 (C=O, $C_6H_3Cl_2$-NH-C_6H_4-CH_2-**CO**ON=C-3); 1720 (N=C, $C_6H_3Cl_2$-NH-C_6H_4-CH_2-COO**N=C**-3); 1625 (C=O, -**C(O)**Mor); 995 (C–N, -C(O)**Mor**); Mor = morpholine ring.<br>**^{13}C NMR** (151 MHz, $CDCl_3$) δ: 175.87 (C_q, $C_6H_3Cl_2$-NH-C_6H_4-CH_2-**CO**ON=C-3, C-1′); 175.17 (C_q, -**C(O)**Mor); 170.55 (C_q, C-3); 144.65 (C_q, C-13); 142.91 x 2 (C_q x 2, **C_6H_3**Cl_2-NH-C_6H_4-CH_2-COON=C-3, C-10′ and C-14′); 137.95 (C_q, **C_6H_3**Cl_2-NH-C_6H_4-CH_2-COON=C-3, C-9′); 130.98 and 130.83 (CH x 2, **C_6H_3**Cl_2-NH-C_6H_4-CH_2-COON=C-3, C-11′ and C-13′); 129.61 (CH, $C_6H_3Cl_2$-NH-**C_6H_4**-CH_2-COON=C-3, C-6′); 128.84 (CH, $C_6H_3Cl_2$-NH-**C_6H_4**-CH_2-COON=C-3, C-7′); 127.98 (CH, $C_6H_3Cl_2$-NH-**C_6H_4**-CH_2-COON=C-3, C-4′); 127.94 (C_q, $C_6H_3Cl_2$-NH-**C_6H_4**-CH_2-COON=C-3, C-8′); 124.30 (C_q, $C_6H_3Cl_2$-NH-**C_6H_4**-CH_2-COON=C-3, C-3′); 124.02 (CH, **C_6H_3**Cl_2-NH-C_6H_4-CH_2-COON=C-3, C-12′); 121.51 (CH, C-12); 118.51 (CH, $C_6H_3Cl_2$-NH-**C_6H_4**-CH_2-COON=C-3, C-5′); 66.94 x 2 (CH_2 x 2, -C(O)**Mor**); 60.42 (CH_2, $C_6H_3Cl_2$-NH-C_6H_4-**CH_2**-COON=C-3, C-2′); 47.32 (C_q, C-17); 46.05 (CH_2, -C(O)Mor), 41.58 (CH_2, -C(O)**Mor**); Mor = morpholine ring.

		[1]**H NMR** (600 MHz, $CDCl_3$) δ: 7.36 and 7.34 (2 x 1H, 2 x s, **C_6H_3**Cl_2-NH-C_6H_4-CH_2-COON=C-$_3$, C-$_{11'}$-H and C-$_{13'}$-H); 7.28 (1H, dd, *J* = 7.5 and 1.1 Hz, $C_6H_3Cl_2$-NH-**C_6H_4**-CH_2-COON=C-$_3$, C-$_{6'}$-H); 7.14 (1H, td, *J* = 7.6 and 1.2 Hz, **C_6H_3**Cl_2-NH-C_6H_4-CH_2-COON=C-$_3$, C-$_{12'}$-H); 7.01 (1H, t, *J* = 8.0 Hz, $C_6H_3Cl_2$-NH-**C_6H_4**-CH_2-COON=C-$_3$, C-$_{5'}$-H); 6.97 (1H, t, *J* = 7.3 Hz, $C_6H_3Cl_2$-NH-**C_6H_4**-CH_2-COON=C-$_3$, C$_{4'}$-H); 6.88 (1H, s, $C_6H_3Cl_2$-**NH**-C_6H_4-CH_2-COON=C-$_3$); 6.57 (1H, d, *J* = 8.0 Hz, $C_6H_3Cl_2$-NH-**C_6H_4**-CH_2-COON=C-$_3$, C-$_{7'}$-H); 5.28 (1H, t, *J* = 3.5 Hz, C-$_{12}$-H); 3.96 (2H, s, $C_6H_3Cl_2$-NH-C_6H_4-**CH_2**-COON=C-$_3$, C-$_{2'}$-H_2); 3.69 – 3.56 (8H, m, -C(O)**Mor**), 3.09 (2H, d, *J* = 11.4 Hz, C-$_{18}$-H_β); 1.44, 1.26, 1.18, 1.07, 1.00, 0.99, 0.91 (7 x 3H, 7 x s, 7 x CH_3 groups); Mor = morpholine ring.<br>**MS/EI** (m/z): 816.95 (21.1%, $M^{\bullet +}$).<br>**Elem. anal.**: for $C_{48}H_{63}Cl_2N_3O_4$ calcd.: C: 70.57%, H: 7.77%, N: 5.14%, found: C: 70.55%, H: 7.79, N: 5.16%.

3. ^{1}H NMR and ^{13}C NMR spectra of the novel DCL-OAO (**4a** - **4d**).

Figure S1. **^{1}H NMR** spectra of conjugate **4a**

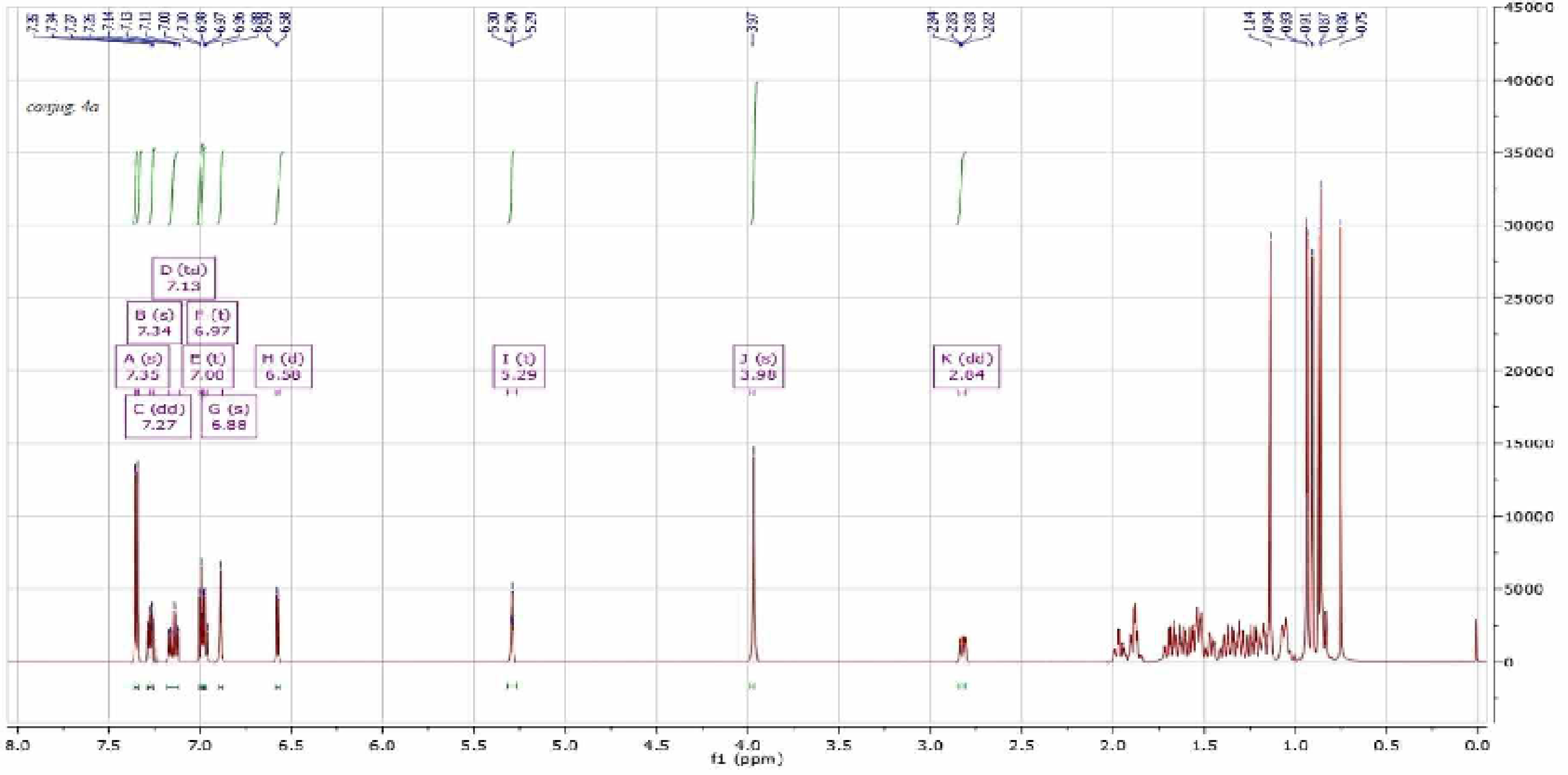

Figure S2. ^{13}**C NMR** spectra of conjugate **4a**

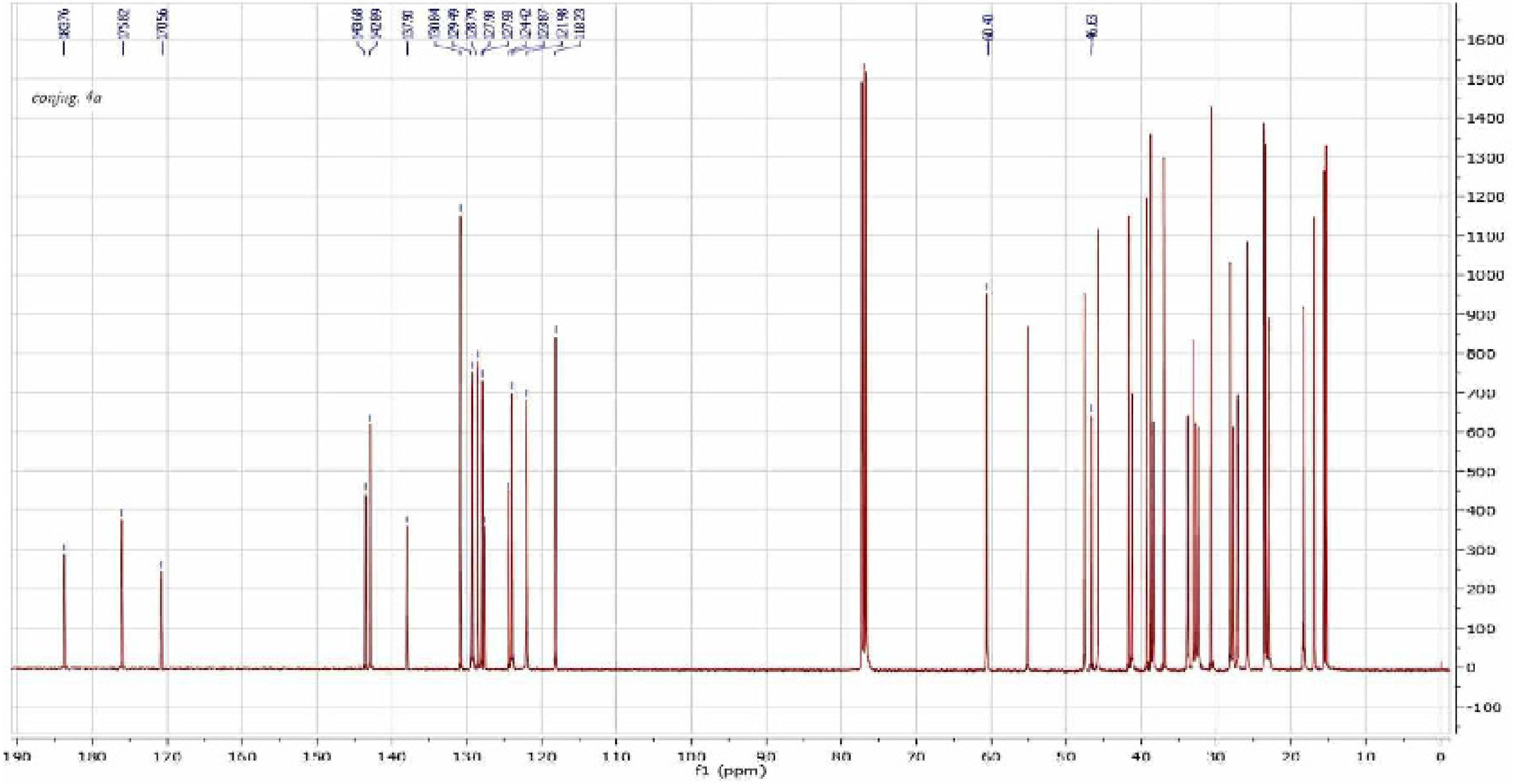

Figure S3. ^{1}**H NMR** spectra of conjugate **4b**

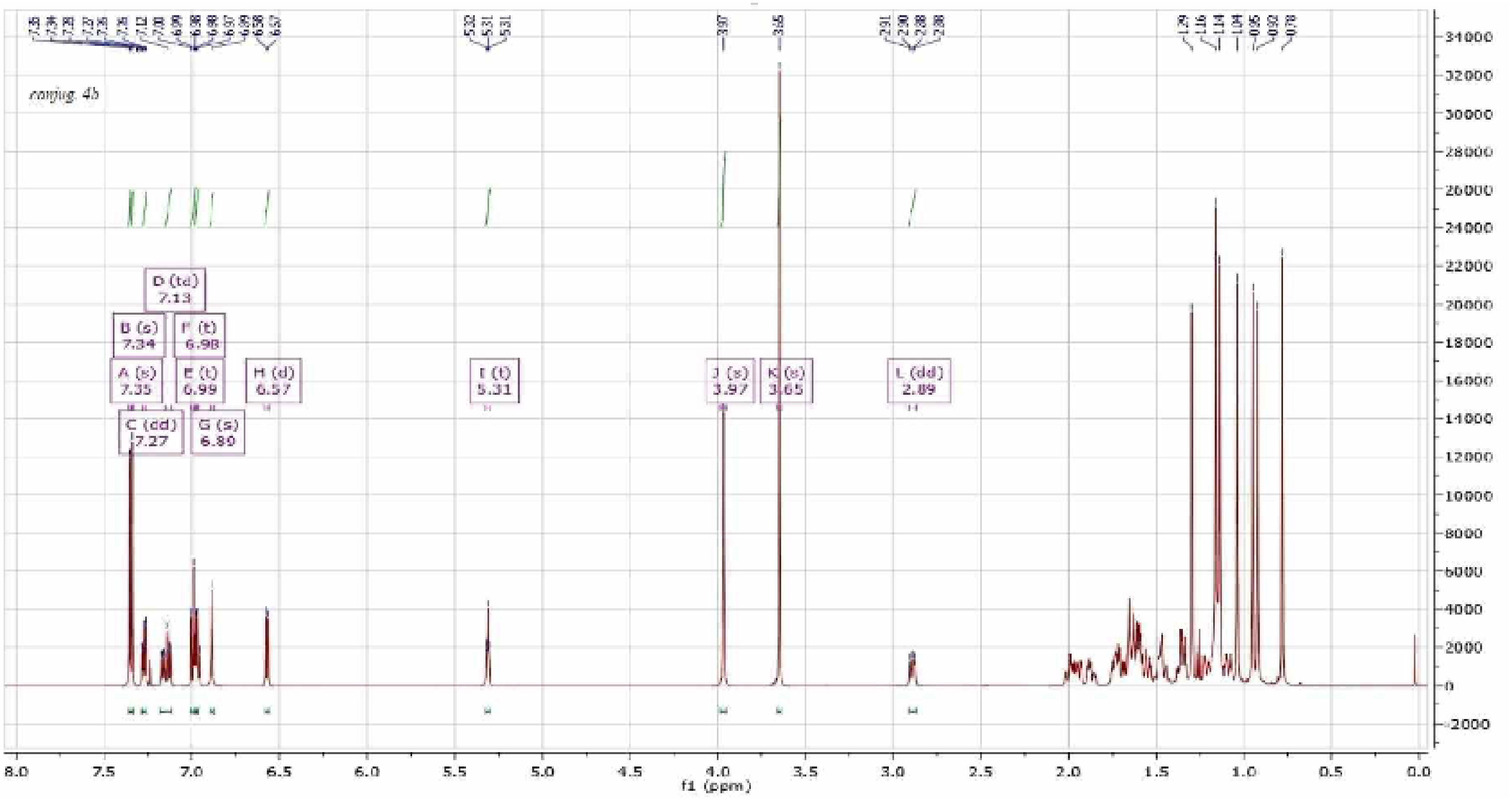

Figure S4. ^{13}**C NMR** spectra of conjugate **4b**

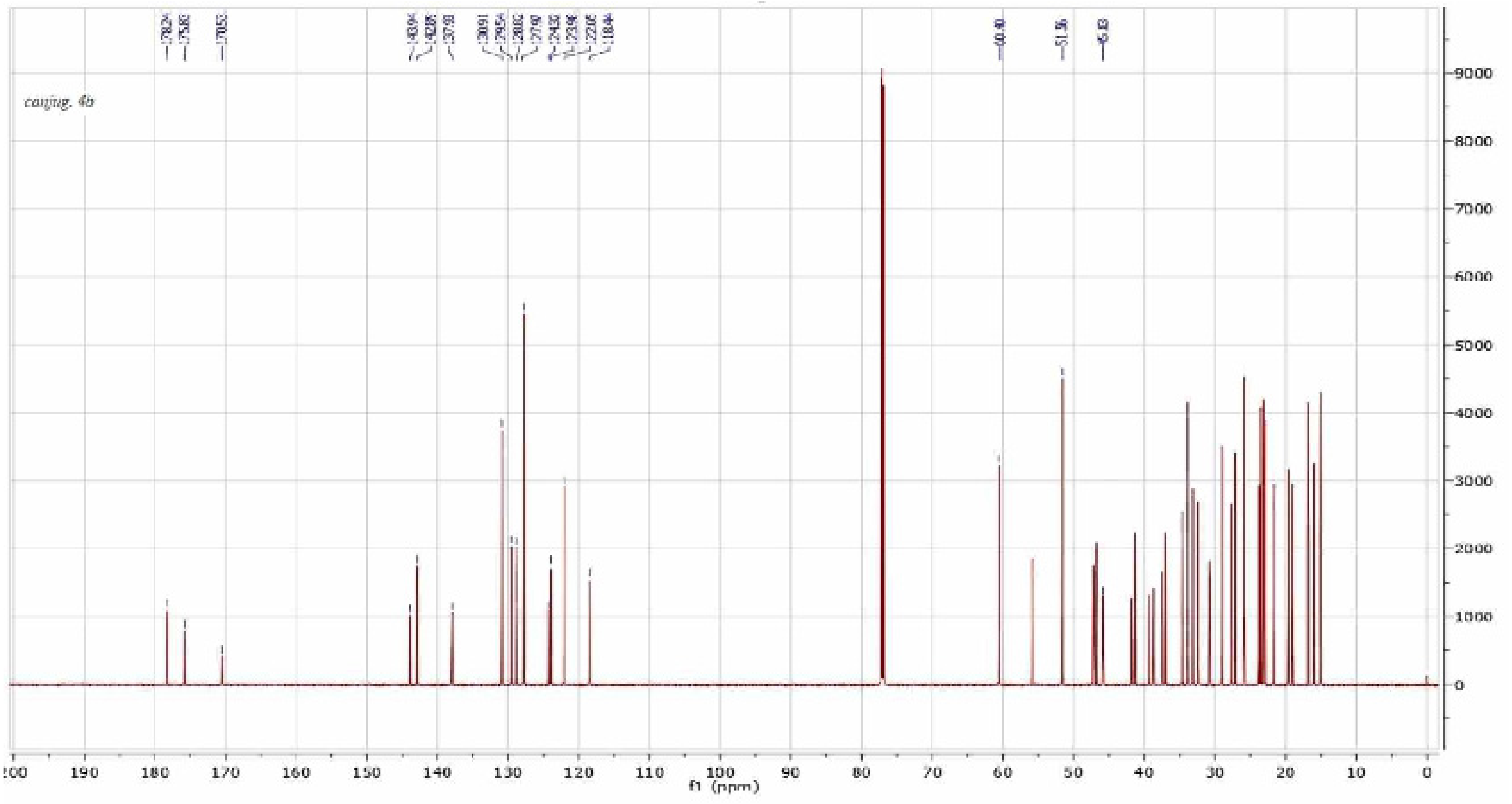

Figure S5. ^{1}**H NMR** spectra of conjugate **4c**

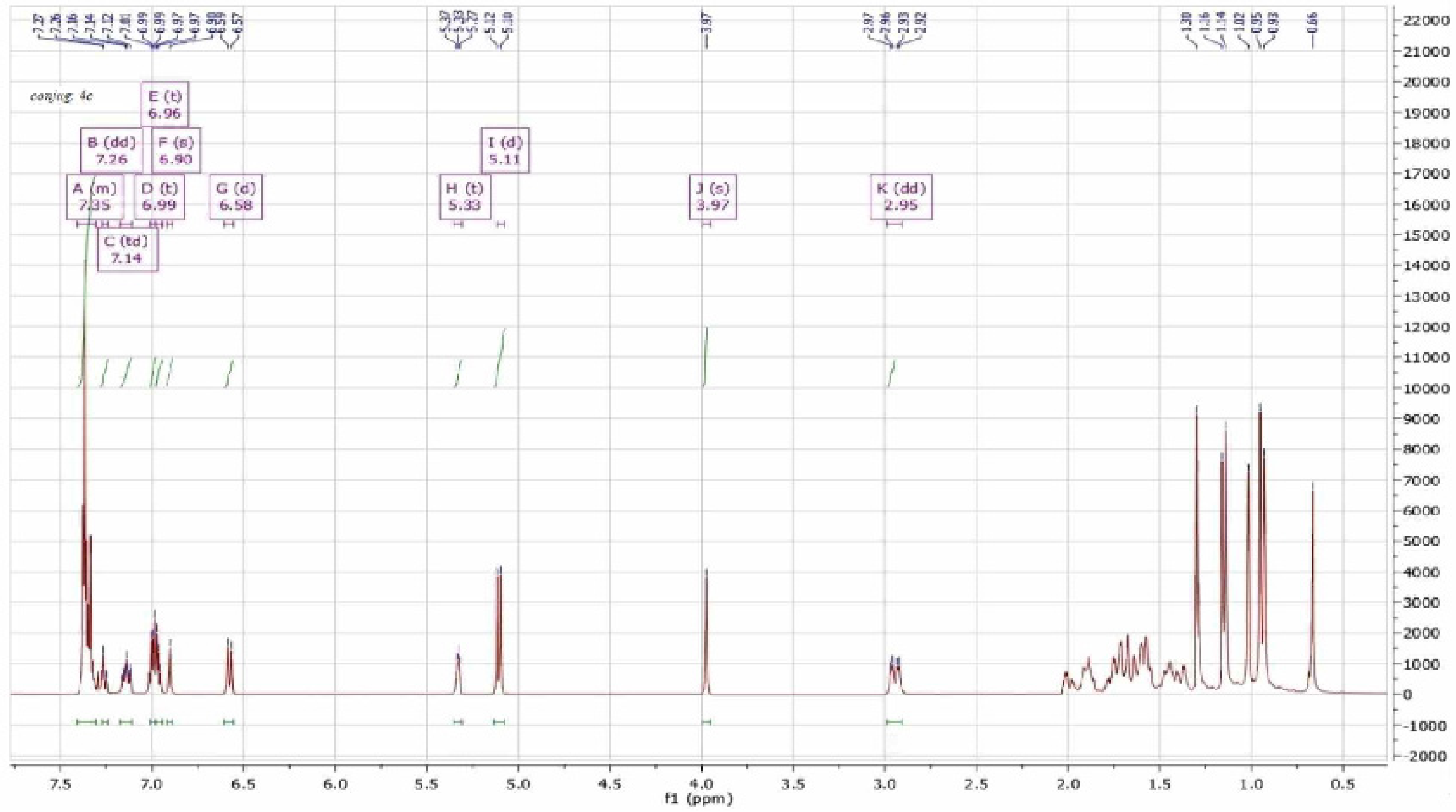

Figure S6. ^{13}**C NMR** spectra of conjugate **4c**

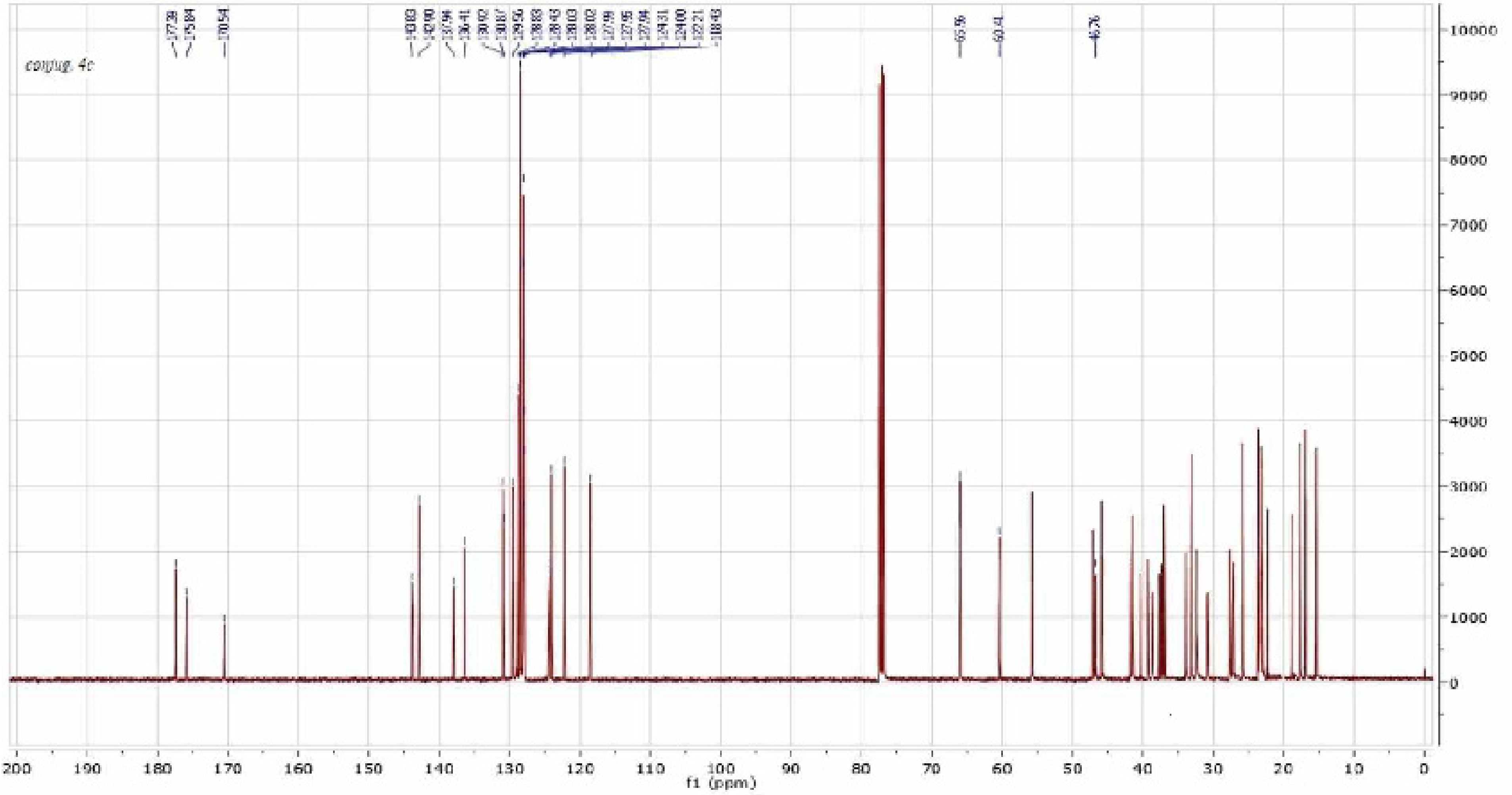

Figure S7. ¹H NMR spectra of conjugate **4d**

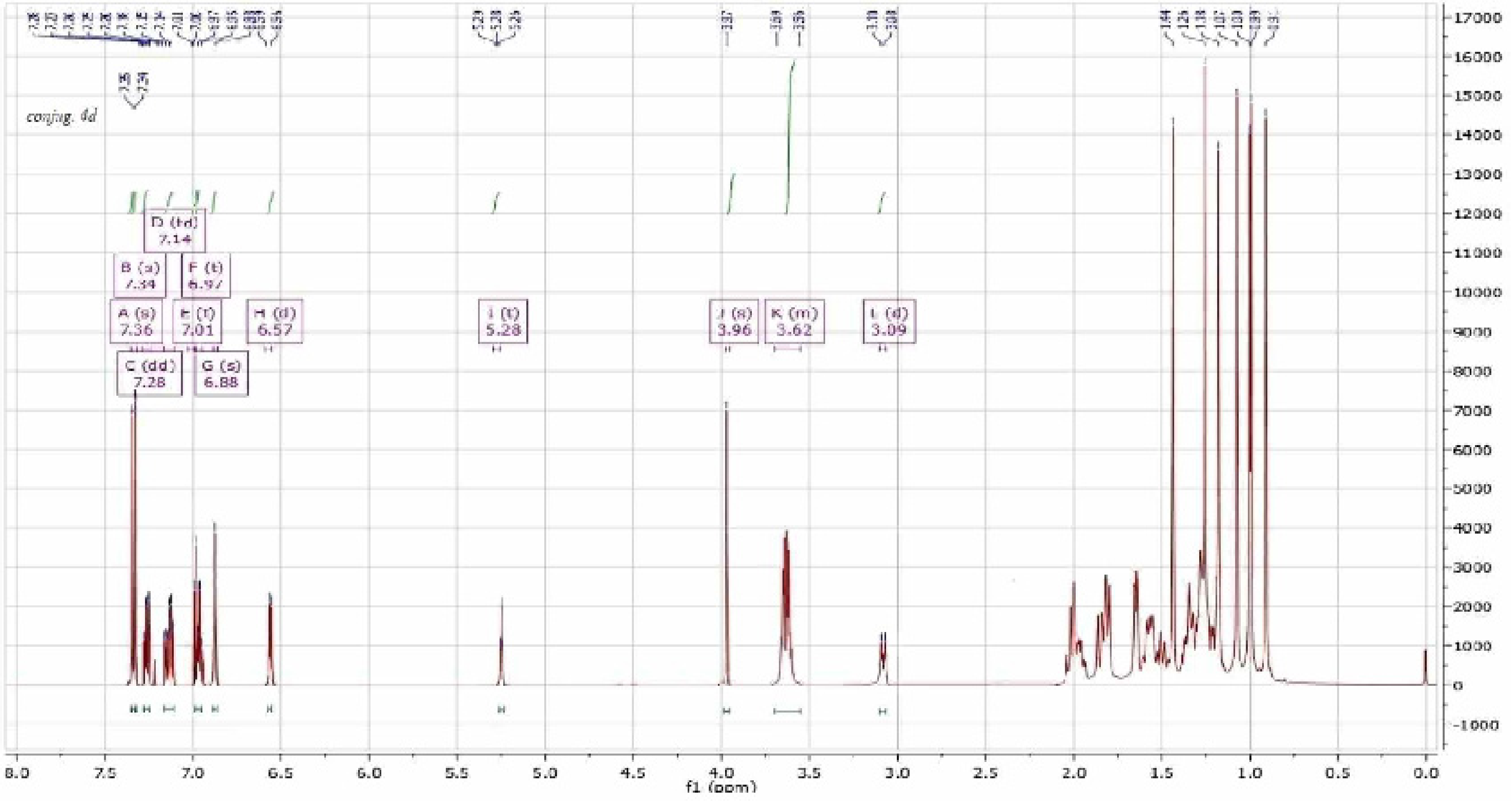

Figure S8. ^{13}**C NMR** spectra of conjugate **4d**

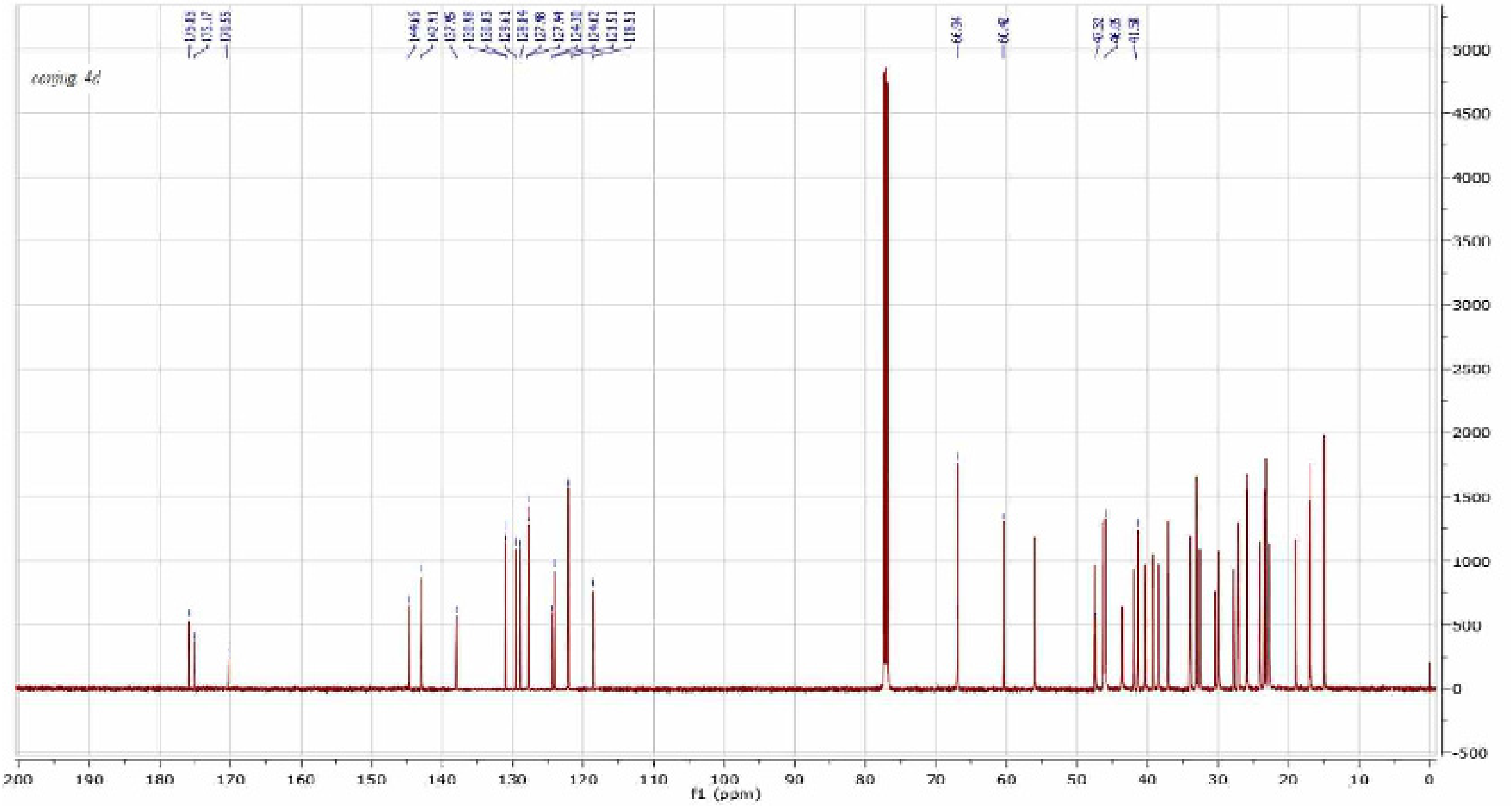

4. Expanded description of the *in silico* studies on ligands (**4c**) and (**4d**) interactions with the Kelch binding domain of the Keap1 protein

4.1. Optimization of ligands and residues involved in hydrogen bond formation

To further explore the geometry of docked ligands in the binding pocket and their interactions with the pocket amino acids, we decide to apply the DFT formalism (B3LYP/6-31G(d,p) approach, ONIOM model 1, **Table S2**) or semiempirical PM6 (ONIOM model 2, **Table S3**)or PM7 (NIOM model 3, **Table S4**) methods including ONIOM (QM:MM) models and to further comparison with the results of docking procedure. In this manner, we optimized only the ligands (high layer). Optimization of the amino acids (low layer) carried out with use of UFF force field [1]. The computations were limited to a 5 Å sphere around the ligand, so the remaining amino acids were treated as frozen. Computations regarding the DFT formalism and (**4c**)–protein complex (**Table S2**) resulted in the elongation of the C=O···H-N contact with Gln530 (Δ = 1.626 Å), while for Ser555 the contact C=O···H-O was not considerably lengthened (Δ = 0.451 Å). The distance between the chlorine atom of (**4c**) and H-N atom of Arg455 turned out to be shorter (Δ = -0.350 Å). By applying the PM6 (**Table S3**) or PM7 (**Table S4**) functionals, we noticed the elongation of all contacts previously detected after the docking procedure. For Arg415, this lengthening was of 0.162 Å (ONIOM model 2) or 0.346 Å (ONIOM model 3), and for Ser555 seemed to be more significant (Δ = 1.416 or 1.461 Å for ONIOM model 2 and 3, respectively). Other contacts underwent a significant elongation during the refinement. Thus, we regarded these bonds as a negligible contact. The elongation of contacts between (**4d**) and particular amino acids under the implementation of DFT formalism, as well as PM6 functional, referred to the N···H-N_{Arg415} (Δ = 0.893 or 0.189 Å, respectively) and C-Cl···H-O_{Ser555} (Δ = 0.407 or 0.166 Å, respectively). However, pyrrolic chlorine interaction involving N-H atom of Arg415 or C-H···$N(H)_{Gly462}$ type of contact were considerably elongated and exceeded the distance of 4 Å making them negligible in this model. On the other hand, the contact between chlorine of (**4d**) and N-H atom of Gln530 was decreased (Δ = -0.732 Å). The distance regarding the same type of contact of benzyl analog (**4c**) involving Gln530 exceeded the value of 4 Å in the ONIOM model 1. Optimization of protonated ligand **4d_H** within the complex using PM7 functional led to an adduct, in which contacts with Arg415, Gly462, and Gln530 turned out to be negligible. Only in the case of Ser555, the long-range contact was elongated (Δ = 0.534 Å).

Based on the resulted data regarding docking protocol, as well as an optimization involving QM:MM models, we can assume that the Arg415 and Gln530 seemed to be crucial in hydrogen bond formation in terms of interactions of analyzed ligands within the cavity of the ligand–protein (4XMB.pdb) complex.

Table S2. Ligand-amino acid contacts (under *d*≤4.0 Å) for the first poses of DCL-OAO derivatives conjugates after ligands optimization with B3LYP/6-31G(d,p) method (ONIOM method, model 1); $RMSD_{complex}$ = 0.430 (**4c**), 0.372 (**4d**), 0.460 (**4d_H**), Å, respectively.

Contact	Contacts Calculated for Docked DCL-OAO Derivatives		
	**4c**	**4d**	**4d_H**
N···H-N_{Arg415}	X	3.885	X
C-Cl···H-N_{Arg415}	3.124	X	X
C-H···$N(H)_{Gly462}$	X	X	3.730
C=O···H-N_{Gln530}	3.895	X	X
C-Cl···H-N_{Gln530}	X	X	X
C=O···H-O_{Ser555}	2.952	X	X
C-Cl···H-O_{Ser555}	X	3.095	3.049
N-H···O_{Tyr572}	X	X	3.708

Table S3. Ligand-amino acid contacts (under *d*≤4.0 Å) for the first poses of DCL-OAO derivatives conjugates after ligands optimization with PM6 method (ONIOM method, model 2); $RMSD_{complex}$ = 0.240 (**4c**), 0.499 (**4d**), 0.515 (**4d_H**), Å, respectively.

Contact	Contacts Calculated for Docked DCL-OAO Derivatives		
	**4c**	**4d**	**4d_H**
N···H-N_{Arg415}	X	3.181	X
C-Cl···H-N_{Arg415}	3.636	X	X
C-H···$N(H)_{Gly462}$	X	X	3.849
C=O···H-N_{Gln530}	X	X	X
C-Cl···H-N_{Gln530}	X	2.875	X
C=O···H-O_{Ser555}	3.917	X	X
C-Cl···H-O_{Ser555}	X	2.854	X
N-H···O_{Tyr572}	X	X	3.714

Table S4. Ligand-amino acid contacts (under *d*≤4.0 Å) for the first poses of OAO–DCL derivatives conjugates after ligands optimization with PM7 method (ONIOM method, model 3); $RMSD_{complex}$ = 0.364 (**4c**), 0.351 (**4d**), 0.535 (**4d_H**), Å, respectively.

Contact	Contacts Calculated for Docked DCL-OAO Derivatives		
	**4c**	**4d**	**4d_H**
N···H-N_{Arg415}	3.945	X	X
C-Cl···H-N_{Arg415}	3.820	X	X
C-H···$N(H)_{Gly462}$	X	X	3.881
C=O···H-N_{Gln530}	X	X	X
C-Cl···H-N_{Gln530}	X	X	X
C=O···H-O_{Ser555}	3.962	X	3.757
C-Cl···H-O_{Ser555}	X	3.222	X
N-H···O_{Tyr572}	X	X	3.768

4.2. The SAPT analysis of ligand-amino acid complexes

Next, we employed the above data for the analysis of interaction energy of docked ligands (**4c**)–(**4d**) additionally with the amino acids involved in the hydrogen bonding or π–π or T–stackings (**Table S5**) using the symmetry-adapted perturbation theory (SAPT) and the *Psi4 1.3.2* software [2] treating the isolated complexes ligand-amino acid as a closed-shell system (as is described in our previously published report [3] and utilizing the recommended jun-cc-pVDZ basis set [4]. Apart from ligand-amino acid total interaction energy calculations, on this account, we considered the following energetic components: the electrostatics, exchange, induction, and dispersion terms as well. The interaction energy of (**4c**) or (**4d**) with Arg415 was almost identical with electrostatics, exchange, induction, and dispersion terms as follows: -5.587 or -9.203, 26.215 or 31.623, -4.238 or -6.071, -19.055 or -18.617 kcal mol^{-1} (for (**4c**) or (**4d**), respectively). In this type of contacts an induction term was dominated; however the hydrogen bod forming between (**4d**) and Arg415 was significantly supported by the electrostatic component. The highest negative total energy SAPT0 (-6.097kcal mol^{-1}) for the interaction with Gln530 was obtained for benzyl derivative (**4c**) compared to its morpholide analog (**4d**). We noticed the following values of electrostatics, exchange, induction, and dispersion terms: -5.798 or -0.207, 3.578 or 0.463, -1.306 or -0.275, -2.571 or -1.339 kcal mol^{-1} (for (**4c**) or (**4d**), respectively). It was clear that the contact of (**4c**) with Gln530 was more electrostatics in its nature. In the case of interaction with Gly462 and Ser555, the highest negative total energy SAPT0 was related with a ligand containing

morpholide moiety (**4d**). Here the dispersion terms seemed to be crucial (in case of Gly462: -1.461 or -3.521 kcal mol^{-1} for (**4c**) or (**4d**), respectively; regarding the Ser555: -7.085 or -2.410 kcal mol^{-1} for (**4c**) or (**4d**), respectively).

During the docking procedure, we observed that the distance between chlorine within (**4c**) or carbocyclic skeleton of (**4d**) and phenolic ring of Tyr334 equaled ca. 4 Å. On this account, surprisingly more negative value of total energy SAPT0 referred to the (**4d**) derivative. Here the dispersion term seemed to be dominated and equaled: -4.323 or -9.248 kcal mol^{-1} for (**4c**) and (**4d**), respectively, as originated from the mutual T–stacking type of ligand-amino acid interaction. Close proximity (ca. 3 Å) of the phenyl ring and the carbonyl group of (**4d**) to the phenolic functionality of Tyr572 influenced on the more negative total SAPT0 interaction energy (in comparison with the (**4c**) analog). It was basically caused by the dispersion terms equaled: -13.420 kcal mol^{-1} (against -4.038 kcal mol^{-1} for (**4c**)–Tyr572 type interaction). For interactions involving (**4c**) and (**4d**) with Phe577, we observed that they were significantly caused by dispersion contribution to the SAPT0 energy that equaled: -1.837 and -10.716 kcal mol^{-1} pointing to the morpholide analog to be more potent (even though the distance between carbonyl group and carbocyclic skeleton within (**4d**) and phenyl ring of amino acid was about 4 Å). It is noteworthy that the closer proximity of chlorophenyl ring within (**4c**) to the phenyl ring of Phe577 (ca. 3 Å) had noticeable impact on the discrepancies in the electrostatics term that equaled: -0.503 and -4.248 kcal mol^{-1}. Obviously, type of interaction between (**4d**) and Phe577 is strongly dependent on both dispersion and the electrostatics terms.

Table S5. Calculated total values of the interaction ligand-amino acid energy [kcal/mol] using the SAPT0 method for docked DCL-OAO derivatives conjugates.

**Amino Acid**	**Calculated Value of Total SAPT0 Energy for Docked OAO–DCL derivatives**	
	**4c**	**4d**
Tyr334	-3.054	-5.375
Arg415	-2.666	-2.269
Gly462	-0.829	-1.555
Gln530	-6.097	-1.359
Ser555	-1.610	-2.648
Tyr572	-4.126	-7.303
Phe577	-1.687	-2.717

4.3. Estimation of the interaction energy

In the next step, we optimized complexes of the docked ligands interacting with the cavity of the 4XMB.pdb protein using a semiempirical approach. Our experiment was carried out as was described in our previously published papers [5]. Implementation of the PM7 Hamiltonian for the ligand–cavity complex optimization (with the distance within of 4 Å, **Table S6**) led to a conclusion that in the case of the (**4c**) derivative, we observed in changes of the N···H-N_{Arg415} (distance decreased to the value of 2.241 Å) and C-Cl···H-N_{Arg415} (Δ = -0.914 Å). Other contacts detected during docking procedure were neglected as their distances exceeded the value of 4 Å. For the morpholide derivative (**4d**) and its protonated analog **4d_H** the changes were quite similar and were related to their interaction with Arg415, Gly462 and Ser555 Considering the nitrogen atom's protonation within the morpholide functionality, we noticed that the greater changes of distances regarding the above-mentioned amino acids were devoted to the C-H···$N(H)_{Gly462}$ type of contact (Δ = -0.400 Å, when compare contacts formed by the (**4d**) and **4d_H** ligands). On the contrary, the smallest changes regarded the C-Cl···H-N_{Arg415} (Δ = -0.040 Å, when compare contacts formed by the (**4d**) and **4d_H** ligands). Furthermore, we focused on assessing enthalpy changes of the interactions of OAO-DCL ligands in the 4XMB.pdb pocket. This evaluation considered values of the final heat of formation (HOF) under standard conditions using the *Mopac 2016* program and its implemented module Mozyme. While analyzing the enthalpy of interactions (-135.64, -158.86 and -142.07 kcal mol^{-1} for the (**4c**), (**4d**) and **4d_H** ligands, respectively), it was evident that morpholide was clearly preferable from the standpoint of interaction enthalpy. The impact of protonation of the morpholide moiety on the HOF values seemed to be insignificant. It is noteworthy that the resulted data agreed with the binding affinity estimation during the docking procedure.

Table S6. Ligand-amino acid contacts (under *d*≤4.0 Å) for the first poses of DCL-OAO derivatives conjugates after ligands optimization with PM7 method; $RMSD_{complex}$ = 0.893 (**4c**), 1.652 (**4d**), 1.244 (**4d_H**), Å, respectively.

**Contact**	**Contacts Calculated for Docked DCL-OAO Derivatives**		
	**4c**	**4d**	**4d_H**
N···H-N_{Arg415}	2.241	2.825	2.691
C-Cl···H-N_{Arg415}	2.560	3.638	3.598
C-H···$N(H)_{Gly462}$	X	3.423	3.023
C=O···H-N_{Gln530}	X	X	X
C-Cl···H-N_{Gln530}	X	X	X
C=O···H-O_{Ser555}	X	X	X
C-Cl···H-O_{Ser555}	X	2.865	2.760
N-H···O_{Tyr572}	X	X	X

References from the Supplementary Materials

1. Rappé, A.K.; Casewit, C.J.; Colwell, K.S.; Goddard, W.A.; Skiff, W.M. UFF, a Full Periodic Table Force Field for Molecular Mechanics and Molecular Dynamics Simulations. *J. Am. Chem. Soc.* **1992**, *114*, 10024–10035, doi:10.1021/ja00051a040.
2. Parrish, R.M.; Burns, L.A.; Smith, D.G.A.; Simmonett, A.C.; DePrince, A.E.; Hohenstein, E.G.; Bozkaya, U.; Sokolov, A.Y.; Di Remigio, R.; Richard, R.M.; et al. Psi4 1.1: An Open-Source Electronic Structure Program Emphasizing Automation, Advanced Libraries, and Interoperability. *J. Chem. Theory Comput.* **2017**, *13*, 3185–3197, doi:10.1021/acs.jctc.7b00174.
3. Czaja, K.; Kujawski, J.; Śliwa, P.; Kurczab, R.; Kujawski, R.; Stodolna, A.; Myślińska, A.; Bernard, M.K. Theoretical Investigations on Interactions of Arylsulphonyl Indazole Derivatives as Potential Ligands of VEGFR2 Kinase. *Int. J. Mol. Sci.* **2020**, *21*, 4793, doi:10.3390/ijms21134793.
4. Parker, T.M.; Burns, L.A.; Parrish, R.M.; Ryno, A.G.; Sherrill, C.D. Levels of symmetry adapted perturbation theory (SAPT). I. Efficiency and performance for interaction energies. *J. Chem. Phys.* **2014**, *140*, 094106, doi:10.1063/1.4867135.
5. Czaja, K.; Kujawski, J.; Kamel, K.; Bernard, M.K. Selected arylsulphonyl pyrazole derivatives as potential Chk1 kinase ligands—computational investigations. *J. Mol. Model.* **2020**, *26*, 1–11, doi:10.1007/s00894-020-04407-3.
